# Application of a new methodology and R package reveals a high burden of healthcare-associated infections (HAI) in Germany compared to the average in the European Union/European Economic Area, 2011 to 2012

**DOI:** 10.2807/1560-7917.ES.2019.24.46.1900135

**Published:** 2019-11-14

**Authors:** Benedikt Zacher, Sebastian Haller, Niklas Willrich, Jan Walter, Muna Abu Sin, Alessandro Cassini, Diamantis Plachouras, Carl Suetens, Michael Behnke, Petra Gastmeier, Lothar H. Wieler, Tim Eckmanns

**Affiliations:** 1 Robert Koch Institute, Berlin, Germany; 2 These authors contributed equally to this work; 3 European Centre for Disease Prevention and Control, Stockholm, Sweden; 4 Charité Universitätsmedizin Berlin, Berlin, Germany

**Keywords:** healthcare-associated infections, burden of disease, Disability-adjusted Life Years, Point prevalence Survey

## Abstract

**Background:**

Healthcare-associated infections (HAIs) pose a major challenge to health systems. Burden of disease estimations in disability-adjusted life years (DALYs) are useful for comparing and ranking HAIs.

**Aim:**

To estimate the number of five common HAIs, their attributable number of deaths and burden for Germany.

**Methods:**

We developed a new method and R package that builds on the approach used by the Burden of Communicable Diseases in Europe (BCoDE) project to estimate the burden of HAIs for individual countries. We used data on healthcare-associated *Clostridioides difficile* infection, healthcare-associated pneumonia, healthcare-associated primary bloodstream infection, healthcare-associated urinary tract infection and surgical-site infection, which were collected during the point prevalence survey of HAIs in European acute-care hospitals between 2011 and 2012.

**Results:**

We estimated 478,222 (95% uncertainty interval (UI): 421,350–537,787) cases for Germany, resulting in 16,245 (95% UI: 10,863–22,756) attributable deaths and 248,920 (95% UI: 178,693–336,239) DALYs. Despite the fact that Germany has a relatively low hospital prevalence of HAIs compared with the European Union/European Economic Area (EU/EEA) average, the burden of HAIs in Germany (308.2 DALYs/100,000 population; 95% UI: 221.2–416.3) was higher than the EU/EEA average (290.0 DALYs/100,000 population; 95% UI: 214.9–376.9). Our methodology is applicable to other countries in or outside of the EU/EEA. An R package is available from https://CRAN.R-project.org/package=BHAI.

**Conclusion:**

This is the first study to estimate the burden of HAIs in DALYs for Germany. The large number of hospital beds may be a contributing factor for a relatively high burden of HAIs in Germany. Further focus on infection prevention control, paired with reduction of avoidable hospital stays, is needed to reduce the burden of HAIs in Germany.

## Introduction

Healthcare-associated infections (HAIs) are associated with an increased risk in morbidity, mortality and excess healthcare costs. It is estimated that 20 to 30% of HAIs in Germany could be preventable primarily through improved adherence to hygiene recommendations and optimisation of procedures [1]. Increasing adherence and changing behaviour of clinical personnel is resource-intensive, and resources for prevention are limited [2]. Estimates of health burden of HAIs are therefore needed to assess their relevance compared with other communicable diseases and help with evidence-based prioritisation. Since a patient with a HAI experiences this alongside the primary reason for being in a healthcare setting, attributing complications and death to a HAI is particularly challenging. 

The European Centre for Disease Prevention and Control (ECDC) published the first estimates of the health burden attributable to HAIs in the European Union and European Economic Area (EU/EEA) in 2016 [3]. It used the same incidence-based approach as the ECDC Burden of Communicable Diseases in Europe (BCoDE) project to estimate the number of disability-adjusted life years (DALYs) due to HAIs. The estimates were based on data from the ECDC point prevalence survey (PPS) of HAIs and antimicrobial use in European acute care hospitals between 2011 and 2012 [4–6]. DALYs are a comprehensive measure to rank the burden of diseases and this way of ranking is more valuable than ranking only on incidence or prevalence. The BCoDE tool assists in calculating the number of DALYs specifically for a number of communicable diseases, including HAIs [7].

In this study, we used a new adapted methodology to estimate the incidence of HAIs, attributable deaths and DALYs in 2011 in Germany for five common types of HAIs: healthcare-associated *Clostridioides difficile* infection (CDI), healthcare-associated pneumonia (HAP), healthcare-associated primary bloodstream infection (BSI), healthcare-associated urinary tract infection (UTI) and surgical site infection (SSI). In addition, we compared our estimates of the burden of HAIs in Germany with estimates for the EU/EEA.

## Methods

### Study population and study design

The German PPS data used in this study were collected in 2011 as part of the ECDC PPS, which was conducted between 2011 and 2012 in 29 EU/EEA countries and Croatia, which has since become the 28^th^ EU Member State [6]. Three datasets were available for our analyses:

(i) The representative German PPS sample consists of 46 hospitals, randomly selected from a list of all German hospitals stratified by hospital size [6]. The sample includes 9,626 patients in total.

(ii) A larger German convenience sample of the same PPS, which is not representative by hospital size, consists of 132 hospitals and 41,539 patients.

(iii) For comparison, we used the ECDC PPS sample, which includes 273,753 patients from 1,149 hospitals in 29 EU/EEA countries in 2011 and 2012 and Croatia [6]. We estimated the burden of HAIs for the ECDC PPS sample using the original data and our adapted methodology, which is implemented in the Burden of Healthcare-associated Infections (BHAI) R package (see below for more details).

The types of HAIs selected for this study were as described by Cassini et al. [3]. The selection was based on prevalence, availability of data and evidence from systematic reviews. HAIs were defined according to EU case definitions [8], with the exception of CDIs, for which we selected a syndrome-based approach.

### Outcome measure

DALYs are a composite measure of years lived with disability (YLDs) and years of life lost (YLLs) accounting for incidence, severity and mortality of the disease simultaneously. This approach enables ranking and comparability of the health burden of different types of HAIs in one metric.

### Outcome trees with transition probabilities

Disease models or outcome trees illustrate progression pathways of HAIs over time, starting with acute infection and ending with either recovery, permanent disability, or death. To take into account all possible health consequences of HAIs, outcome trees were developed based on 13 systematic reviews of the literature [9,10]. Attributable transition probabilities were extracted from the literature to avoid overestimation of the health burden due to comorbidities, which is a particular risk in patients with an HAI. Health outcomes were related to each other by a transition probability and each outcome included a duration and a disability weight [9,11]. We used the same outcome trees with transition probabilities and disability weights, which were used by Cassini et al.. Detailed descriptions of the outcome trees can be found in the supplement of Cassini et al. or within the ECDC BCoDE toolkit, in which the disease model parameters are described in detail [3,7]. The systematic reviews and results of meta-analyses were published in a separate document [9].

### Workflow of the Burden of Healthcare-Associated Infections (BHAI) methodology

**Figure 1:**
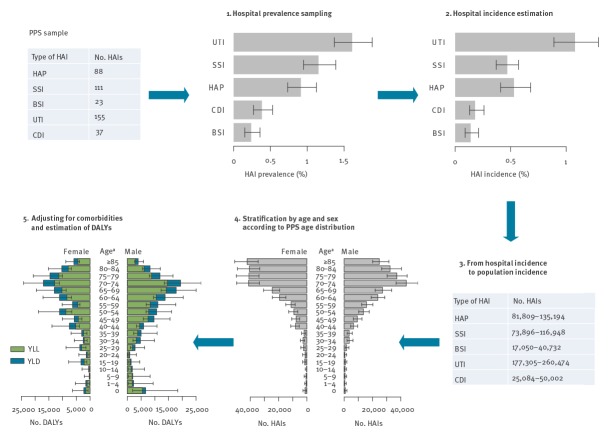
Overview of the workflow of the estimation of the burden of healthcare-associated infections implemented in the BHAI R package

BHAI: Burden of Healthcare-Associated Infections; BSI: healthcare-associated primary bloodstream infection; CDI: healthcare-associated *Clostridioides difficile* infection; DALY: disability-adjusted life year; HAI: healthcare-associated infection; HAP: healthcare-associated pneumonia; No.: Number of; PPS: point prevalence survey; SSI: surgical site infection; UTI: healthcare-associated urinary tract infection; YLD: years lived with disability; YLL: years of life lost.

^a ^Age in years.

Our approach was implemented by the BHAI R package, which performs a predefined number (default: 1,000) of Monte Carlo simulations (Figure 1).

The error bars indicate the 95% uncertainty intervals.

#### Step 1: estimation of hospital prevalence of healthcare-associated infections

For each type of HAI, hospital prevalence (P) was estimated using the observed number of patients with an HAI and the total number of patients in the PPS. Prevalence was calculated separately for the German representative and convenience samples. Uncertainties were taken into account by sampling from a mixture of two beta distributions, which form the exact mid-P-Clopper–Pearson confidence interval (CI) for proportions. The use of this mixed distribution gives uncertainty intervals (2.5% and 97.5% quantiles) that accurately reflect exact CIs.

#### Step 2: estimation of hospital incidence of healthcare-associated infections using prevalence

We estimated hospital incidence (I) from P for each type of HAI using a modification of the Rhame−Sudderth formula: I = P × LA/LOI, where LA is the mean length of stay and LOI is the length of infection [12]. The average LA of all patients in the survey was calculated from data of participating hospitals, based on the number of discharges and patient days in the previous year [13]. The modified Rhame−Sudderth formula defines LOI as the total time of infection. Often, only the time from infection onset until the date of survey (LOI_pps_) is collected. Estimators of LOI that have been used in the past are the median or mean of LOI_pps_, which are approximations for the true LOI. We used the Grenander estimator which was employed in 2018 to estimate the number of HAIs in the EU/EEA [14,15]. In this method, the distributions of LOI_pps_ were estimated from the PPS data assuming monotonicity. This assumption is justified by the timeline of occupancy of a hypothetical bed: There will always be more patients on the first day than on the second day of hospitalisation, and more on the second day than the third day, etc. The average proportion of patients with a HAI on the day of the PPS was derived from the following distribution: Probability(LOI_pps_ = 1). The expected LOI of a HAI was then estimated as 1/Probability(LOI_pps_ = 1). One caveat of this method is that it requires a large sample size. However, Willrich et al. showed that the mean, which is biased but more robust with small samples, performed well in most applications [14]. Our methodology therefore used a weighted sum of the mean of LOI_pps_ and the Grenander estimator using the sigmoid function. This allowed the influence of the Grenander estimator to become larger, and therefore the estimates were more accurate with larger sample sizes. Simulations that analyse the robustness of the LOI estimates for different sample sizes using this new approach are summarised in Supplementary Figure S1. BHAI takes into account the uncertainty about the LOI estimate using bootstraps in each Monte Carlo simulation. The larger German PPS convenience sample was used for more reliable estimation of LOIs. The LOIs used for computations are displayed in Supplementary Figure S2.

#### Step 3: from hospital incidence to population incidence of healthcare-associated infections

Hospital incidences of HAIs per patient were extrapolated to the whole population by multiplying with the number of hospital discharges in Germany. The population data and number of hospital discharges for 2011 were obtained from the Eurostat database (http://ec.europa.eu/eurostat/) for all countries following the approach of Cassini et al. [3,13].

#### Step 4: stratification by age and sex according to point prevalence survey age distribution

After estimating the total annual number of cases in the previous steps, BHAI was used to estimate the number of attributable deaths and DALYs. For DALY calculations, HAIs distributed according to age and sex were needed to account for different life expectancies. For each type of HAI, the age and sex distributions were obtained from the ECDC PPS. With smaller country-wide PPS, the stratified number of cases may exhibit many empty strata (without cases). We used Bayesian analysis to include prior knowledge about the distribution and combined this information with the evidence from the observed data in a posterior distribution of the true parameters.

The posterior distribution was a multinomial likelihood with a Dirichlet prior distribution, which is parameterised by prior weights. The estimation of the health burden from the German PPS sample was conducted by applying the number of cases from the German convenience sample as prior weights. This distribution also contained empty strata, although fewer than in the German PPS sample. Since all prior weights had to be > 0, the BHAI R package added a small pseudocount to all strata: 0.001 × sum(prior weights). In each Monte Carlo simulation, a sample was drawn from the posterior distribution, which incorporated uncertainty about the age and sex distribution. The sampled age and sex probability distribution was then multiplied with the overall annual number of cases to calculate the stratified number of cases for each type of HAI.

#### Step 5: adjusting for different life expectancies

The McCabe score, documented in the ECDC PPS between 2011 and 2012, gave a reference point for the life expectancy of a patient according to severity of underlying disease. It allowed patients to be stratified in three groups according to whether the underlying disease was (i) non-fatal (normal life expectancy), (ii) ultimately fatal (average expected life expectancy of 3 years), or (iii) rapidly fatal (average expected life expectancy of 0.5 years). German PPS data were collected using the unit-based (‘light’) protocol of the ECDC PPS, for which McCabe scores are not recorded [6]. We applied the McCabe score distribution of the ECDC PPS to Germany (i.e. cumulative data from all countries that collected McCabe scores with the standard protocol), assuming that the McCabe score distribution in Germany would be comparable to that of the entire EU/EEA. In the BHAI R package, we calculated a probability distribution of McCabe scores for each HAI, age and sex stratum and distributed cases by multiplication with the number of estimated cases in each simulation. In each Monte Carlo simulation, parameters of disease outcome trees, i.e. disability weights, durations and transition probabilities, were sampled for the whole population. The number of fatalities attributable to each type of HAI was calculated by multiplying the number of cases with the probability of death. The number of DALYs was calculated with respect to the remaining life expectancy in each stratum.

### Validity of estimates obtained with the Burden of Healthcare-Associated Infections (BHAI) method

We simulated ECDC PPS data ranging from 1,000 to 200,000 surveyed patients (the latter being approximately equal to the size of the ECDC PPS sample). This allowed us to compare the sampling approach implemented in the BHAI R package with the approach of the previous burden estimation using the BCoDE toolkit [3,7]. In each simulation, given the number of surveyed patients, the prevalence for each type of HAI from the overall ECDC PPS sample was used to simulate the number of cases of each type of HAI. These were then randomly distributed to strata according to the age, sex and McCabe distributions of the ECDC PPS. The length of the HAIs was sub-sampled according to the number of HAIs in each simulation, while the mean LA was kept fixed to the estimate from the full sample. In order to evaluate the effect of the sample size on the estimation, the results from the simulated data were then compared with the results from the ECDC PPS sample.

### Availability

Our method was implemented by the BHAI R package, which is freely available from https://cran.r-project.org/ and as a Supplement to this paper (Supplement S1). The data and analysis code used for calculating the German and EU/EEA burden of HAIs are attached to the package, which can be used to reproduce the analysis and the results described in this study.

### Ethical statement

This study was based on health information collected and published within the ECDC PPS and did not require informed consent from participants. Reported infectious disease data were provided in aggregated format by specific age and sex strata, without personal identifiers.

## Results

### Burden of healthcare-associated infections in Germany

**Figure 2:**
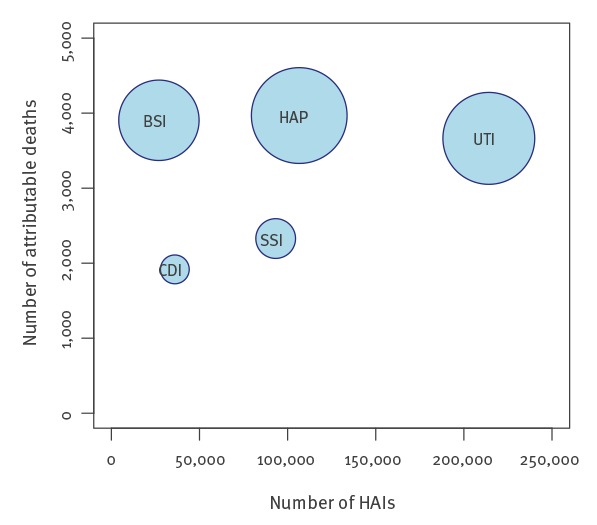
Annual number of healthcare-associated infections plotted against the annual number of attributable deaths for five types of healthcare-associated infections^a^, Germany, 2011

BSI: healthcare-associated primary bloodstream infection; CDI: healthcare-associated *Clostridioides difficile* infection; DALY: disability-adjusted life year; HAI: healthcare-associated infection; HAP: healthcare-associated pneumonia; SSI: surgical site infection; UTI: healthcare-associated urinary tract infection.

^a^ Data are based on the 2011 German point prevalence study sample.

Using German PPS data from 2011, we estimated that there were 478,222 (95% UI: 421,350–537,787) new cases of the selected types of HAIs, and 16,245 (95% UI: 10,863–22,756) attributable deaths in Germany in that year (Table 1). The health burden of acute HAIs and sequelae amounted to a median of 248,920 (95% UI: 178,693–336,239) DALYs, 59,076 (95% UI: 40,263–84,578) YLDs and 190,245 (95% UI: 131,301–264,573) YLLs per year. Note that the sum of the median YLDs and YLLs does not exactly equal the median DALYs. This is because the sum of the median of two distributions (i.e. YLDs and YLLs for all 1,000 simulations) is not necessarily equal to the median of the sum of the distributions (i.e. DALYs calculated as YLD + YLL for each simulation). The relationship between incidence, mortality and DALYs is illustrated in Figure 2, which gives detailed information about the different impacts of HAIs on population health. HAP and BSI accounted for 51% (127,858 DALYs/248,920 DALYs) of the burden of HAIs, but accounted for only 28% (133,562 cases/478,222 cases) of the estimated number of HAIs (Figure 2). UTIs accounted for 45% (214,150 cases/478,222 cases) of the cases, but for 27% (66,701 DALYs/248,920 DALYs) of DALYs. Estimates of the length of the HAIs varied from 9 to 15 days depending on the type of HAI (Supplementary Figure S2).

**Table 1:** Annual burden of five types of healthcare-associated infections, German point prevalence survey sample, Germany, 2011

Type of HAI	Sample	Number of HAIs Point estimate^a^ (95% UI)	Number of attributable deaths Point estimate^a^ (95% UI)	Number of DALYs Point estimate^a^ (95% UI)	Number of YLLs Point estimate^a^ (95% UI)	Number of YLDs Point estimate^a^ (95% UI)
**HAP**	German PPS	106,586 (83,618–137,476)	3,968 (1,107–8,164)	69,508 (34,042–117,232)	41,306 (11,475–84,483)	27,539 (16,528–42,824)
**SSI**	German PPS	93,222 (75,369–114,241)	2,328 (1,888–2,882)	28,842 (23,313–35,303)	28,376 (22,983–34,714)	452 (352–580)
**BSI**	German PPS	26,976 (16,520–42,252)	3,905 (2,004–6,987)	58,350 (30,940–104,227)	49,578 (25,499–90,816)	8,787 (4,463–16,609)
**UTI**	German PPS	214,150 (175,086–253,524)	3,664 (1,462–7,533)	66,701 (27,890–128,543)	44,871 (18,043–92,915)	20,243 (8,095–40,522)
**CDI**	German PPS	36,002 (25,108–49,934)	1,917 (112–4,547)	20,890 (2,023–49,443)	19,937 (1,166–47,973)	977 (172–2,125)
**All**	German PPS	478,222 (421,350–537,787)	16,245 (10,863–22,756)	248,920 (178,693–336,239)	190,245 (131,301–264,573)	59,076 (40,263–84,578)

BSI: healthcare-associated primary bloodstream infection; CDI: healthcare-associated *Clostridioides difficile* infection); DALY: disability-adjusted life year; HAI: healthcare-associated infection; HAP: healthcare-associated pneumonia; PPS: point prevalence survey; SSI: surgical site infection; UI: uncertainty interval; UTI: healthcare-associated urinary tract infection; YLD: years lived with disability; YLL: years of life lost.

^a^ This is the median.

The diameter of the bubbles is proportional to the annual numbers of DALYs, Germany, 2011.

From the larger German convenience sample, we estimated that there were 513,729 (95% UI: 473,840–556,654) HAIs per year, and 19,672 (95% UI: 13,921–26,310) attributable deaths, leading to 290,228 (95% UI: 215,305–372,734) DALYs, 65,988 (95% UI: 46,856–89,194) YLDs and 224,939 (95% UI: 160,973–298,122) YLLs (Supplementary Table S1, Supplementary Figure S3 and S4) in 2011.

In both the German convenience and the representative PPS samples, 4.1%  of patients in hospital (394 patients with HAI/9,626 patients and 1,688 patients with HAI/41,539 patients for each type of sample respectively) had at least one of the five types of HAIs. The difference in disease burden found between the convenience and PPS samples was mainly due to an increased prevalence of HAPs (1.0%; 424 patients with HAP/41,539 patients) and BSIs (0.34%; 142 patients with BSI/41,539 patients) in the German convenience sample.

### Comparison to other communicable diseases

From the German PPS sample, we estimated that the total burden of the five HAIs in Germany was 308.2 DALYs per 100,000 population. In contrast, a previous study using the BCoDE methodology estimated that the burden of four other communicable diseases (influenza, *Salmonella* spp., hepatitis B and measles) in Germany was only 39.4 DALYs per 100,000 population (Supplementary Table S2) [16].

### Comparison to the European Union/European Economic Area

From the ECDC PPS conducted between 2011 and 2012, we estimated the burden of HAIs for the whole EU/EEA using the adapted BHAI method. We found that there were 2,365,466 (95% UI: 2,306,018–2,427,774) HAIs, 77,483 (95% UI: 51,502–106,982) attributable deaths and 1,465,822 (95% UI: 1,086,252–1,905,451) DALYs in the EU/EEA (Supplementary Table S3).

**Figure 3:**
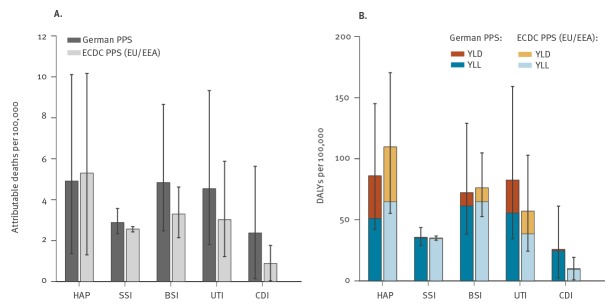
Annual burden of five types of healthcare-associated infections with (A) attributable deaths per 100,000 population and (B) disability-adjusted life years per 100,000 population, Germany, EU/EEA, 2011–2012

BSI: healthcare-associated primary bloodstream infection; CDI: healthcare-associated *Clostridioides difficile* infection; DALY: disability-adjusted life year; ECDC: European Centre for Disease Control and Prevention; EU/EEA: European Union/European Economic Area; HAI: healthcare-associated infection; HAP: healthcare-associated pneumonia; PPS: point prevalence survey; SSI: surgical site infection; UI: uncertainty interval; UTI: healthcare-associated urinary tract infection; YLD: years lived with disability; YLL: years of life lost.

**Figure 4:**
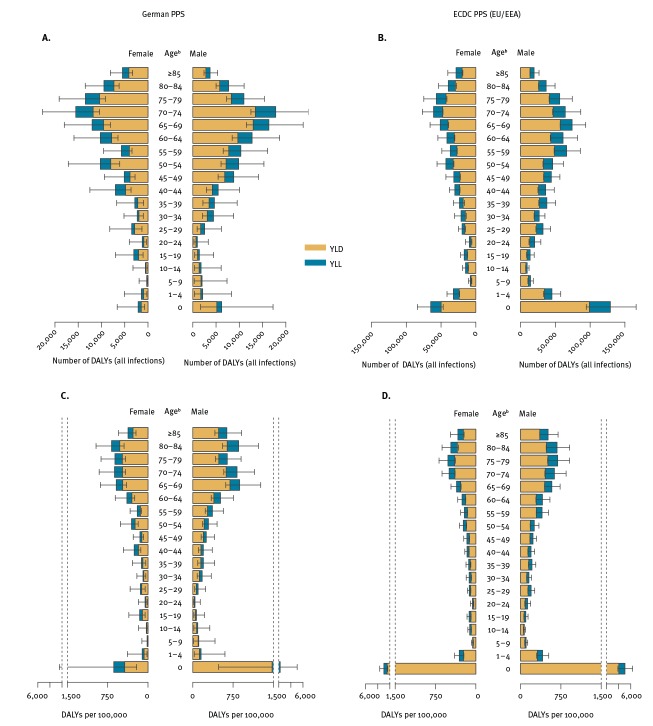
Total annual burden of five types of healthcare-associated infections^a^ in Germany (left) and EU/EEA (right), stratified by age and sex and within each stratum the (A-B) total number of DALYs and (C-D) DALYs per 100,000 population, Germany, EU/EEA, 2011–2012

DALY: disability-adjusted life year; ECDC: European Centre for Disease Control and Prevention; EU/EEA: European Union/European Economic Area; PPS: point prevalence survey.

^a ^The five healthcare-associated  infections comprise healthcare-associated *Clostridioides difficile* infection, healthcare-associated pneumonia, healthcare-associated primary bloodstream infection, healthcare-associated urinary tract infection and surgical site infection.

^b^ Age in years.

Rates per 100,000 population are presented in Table 2 and allow for comparison of the three different samples. The incidence of HAIs in Germany (German PPS representative sample) was 1.27 (95% UI: 1.14–1.43) times higher than the EU/EEA (ECDC PPS sample). The estimated number of attributable deaths per 100,000 population and DALYs per 100,000 population were 1.29 (95% UI: 0.78–2.18) and 1.06 (95% UI: 0.68–1.67) times higher in Germany (German PPS representative sample) than in the EU/EEA. This difference was even larger when comparing rates obtained from the German convenience sample with EU/EEA estimates. There was a 1.36 (95% UI: 1.25–1.47) higher incidence of HAIs, 1.58 (95% UI: 0.95–2.66) higher attributable mortality and 1.24 (95% UI: 0.83–1.84) higher DALY rate in Germany than in the EU/EEA. Comparisons of the rates obtained for Germany (German representative and convenience PPS samples) to the EU/EEA estimates (ECDC PPS sample) are given in Figures 3 and 4 and in Supplementary Figures S5 and S6.

**Table 2:** Annual burden per 100,000 population of five types of healthcare-associated infections, German PPS sample, German convenience sample and ECDC PPS sample, Germany, EU/EEA, 2011–2012

Annual burden measure	Sample	HAP Point estimate^a^ (95% UI)	UTI Point estimate^a^ (95% UI)	BSI Point estimate^a^ (95% UI)	SSI Point estimate^a^ (95% UI)	CDI Point estimate^a^ (95% UI)	All Point estimate^a^ (95% UI)
HAIs per 100,000	German PPS	132.0 (103.5–170.2)	265.1 (216.8–313.9)	33.4 (20.5–52.3)	115.4 (93.3–141.4)	44.6 (31.1–61.8)	592.1 (521.7–665.8)
	German convenience	162.3 (137.5–190.7)	228.7 (200–260.7)	52.7 (42–66.9)	146.9 (126.5–167.8)	44.5 (35.6–55.4)	636.1 (586.7–689.2)
	ECDC PPS (EU/EEA)	143.7 (136.9–150.8)	174.7 (166.3–182.4)	22.2 (20–25.1)	111.3 (105.4–116.6)	16.0 (14.2–18.3)	467.9 (456.2–480.2)
Attributable deaths per 100,000	German PPS	4.9 (1.4–10.1)	4.5 (1.8–9.3)	4.8 (2.5–8.7)	2.9 (2.3–3.6)	2.4 (0.1–5.6)	20.1 (13.4–28.2)
	German convenience	6.1 (1.4–11.7)	3.9 (1.6–8)	7.9 (4.7–11.8)	3.7 (3.2–4.2)	2.5 (0.1–5.3)	24.4 (17.2–32.6)
	ECDC PPS (EU/EEA)	5.3 (1.3–10.2)	3.0 (1.2–5.9)	3.3 (2.1–4.6)	2.6 (2.4–2.7)	0.9 (0–1.8)	15.3 (10.2–21.2)
DALYs per 100,000	German PPS	86.1 (42.1–145.1)	82.6 (34.5–159.2)	72.2 (38.3–129)	35.7 (28.9–43.7)	25.9 (2.5–61.2)	308.2 (221.2–416.3)
	German convenience	103.4 (51.5–166.5)	69.5 (29.9–127.7)	113.5 (72.2–166)	45.0 (38.8–51.3)	26.5 (2.5–55.6)	359.3 (266.6–461.5)
	ECDC PPS (EU/EEA)	109.8 (55.3–170.5)	57.1 (24.3–102.9)	76.2 (52.6–104.8)	35.1 (33.3–36.8)	10.0 (0.9–19.2)	290.0 (214.9–376.9)

BSI: healthcare-associated primary bloodstream infection; CDI: healthcare-associated *Clostridioides difficile* infection; DALY: disability-adjusted life year; ECDC: European Centre for Disease Prevention and Control; EU/EEA: European Union/European Economic Area; HAP: healthcare-associated pneumonia; PPS: point prevalence survey; SSI: surgical site infection; UTI: healthcare-associated urinary tract infection.

^a^ This is the median.

For all five HAIs, attributable deaths amount to 20.1 per 100,000 population (95% UI: 13.4–28.2) and DALYs to 308.2  per 100,000 population (95% UI: 221.2–416.3).

The error bars indicate the 95% UI.

The error bars indicate the 95% uncertainty intervals.

### Burden of Healthcare-Associated Infections (BHAI) methodology and R package

The new methodology for the estimation of HAIs presented in this study was implemented in the R package BHAI (openly available from https://cran.r-project.org/). It was evaluated using a simulated PPS with sample sizes ranging from 1,000–200,000. This evaluation demonstrates the usability of BHAI for the estimation of the burden of HAIs for large and small PPS samples (Supplementary Figure S7).

## Discussion

In this study, we estimated the burden of HAIs in Germany expressed in DALYs for the first time, based on point prevalence data from 2011. By considering incidence and severity of the disease simultaneously, we provided a more comprehensive view on the burden of HAIs than previously available. This allowed for comparing and ranking the burden of HAIs and other diseases in Germany.

The annual number of HAIs in Germany was previously estimated between 400,000–600,000 cases and 10,000–15,000 attributable deaths per year. These estimates were based on data from the German PPS studies (NIDEP-1) from 1994 [17,18]. In the ECDC PPS, the annual number of HAIs in Germany in 2011 was estimated at between 321,321–1,025,716 [6]. It is important to note that different approaches and methods were used in these studies.

In this study, we estimated the numbers of cases for five types of HAI, associated fatalities and their health burden. These five types of HAI made up 79% of all HAIs in the ECDC PPS sample, and the numbers of HAIs in our study therefore do not represent the total number of HAIs in the population. Nevertheless, our estimates of the number of HAIs lie within the range of previous estimates, although the median estimates of the number of attributable deaths were higher.

In our study, BSIs had the lowest incidence of the five types of HAI, but was responsible for a high number of DALYs due to its high attributable mortality. On the other hand, UTI, despite its high incidence, caused a comparable health burden since its attributable mortality was considerably lower. While, of all cases, the fatality rate of primary or secondary BSIs was 9–20%, 5–20% of patients with UTI developed a secondary bacteraemia or urosepsis, which then led to death in 9–20% of the cases [9,10]. These shifts in ranking illustrate the importance of a comprehensive approach such as the burden estimation based on DALYs.

The health burden of HAIs was substantially higher than the burden of other communicable diseases. For instance, the burdens of HAP (69,508 DALYs) and UTI (66,701 DALYs) were more than twice as large in comparison, and the burden of BSI (58,350 DALYs) was slightly less than twice as much as the burden of influenza (33,116 DALYs) in Germany (Supplementary Table S2) [16]. The burden of all five considered HAIs in Germany was 308.2 (95% UI: 221.2–416.3) DALYs. This was also higher than the burden of 31 selected infectious diseases in the EU/EEA, which was estimated to be 273 (95% UI: 249–299) DALYs per 100,000 population using the BCoDE methodology [19]. Comparisons of our estimates of the number of DALYs with those of the Global Burden of Disease (GBD) project must be conducted with caution, since the latter are derived using a prevalence-based approach [20]. In addition, the burden of HAIs is not presented within the GBD project, but is instead attributed to the underlying disease. However, a relative and rough comparison of the burden of HAIs with GBD estimates for other diseases is presented in the Supplementary Table S4 [20,21].

### Comparison of Germany and the European Union/European Economic Area

To ensure comparability, we estimated the burden of the five types of HAIs in the EU/EEA with the same BHAI methodology and R package. The number of HAIs, attributable deaths and DALYs (YLDs and YLLs) per 100,000 population was overall higher in Germany than in the EU/EEA average (Table 2, Supplementary Table S1 and S3, Figure 3, Supplementary Figure S5 and S6). Since the hospital prevalence of HAIs in Germany in 2011 was considerably lower than the EU/EEA average from the ECDC PPS between 2011 and 2012, we initially expected a lower health burden of HAIs in Germany [6]. However, extrapolating from the *hospitalised* to the *general* population (see Methods, workflow step 3–5), the burden of HAIs in Germany was higher than the EU/EEA average. Burden of disease is a measure based on the general population and thus the number of hospitalised patients – which extrapolates the incidence per patient to the general population – is a major factor influencing the burden of HAIs in the general population. Germany has the highest number of curative beds in Europe and the second largest number of hospitalised patients per 1,000 population among 34 Organisation for Economic Co-operation and Development (OECD) countries [22–24]. Hygiene and infection prevention and control measures affect HAI occurrence among hospitalised patients. Germany’s relatively low prevalence among hospitalised patients may be partly explained by the generally good hygiene and infection prevention and control measures in German hospitals. However, since there are so many hospitalised patients, their effectiveness may be diluted when calculating the burden of HAIs per general population. This becomes clear when comparing the incidence within the hospitalised population to the incidence per general population. In accordance with HAI prevalence, the incidence of HAIs within the hospitalised population was lower for Germany – 2.40 (95% UI: 2.12–2.70) per 100 patients – than for the EU/EEA – 2.83 (95% UI: 2.75–2.90) per 100 patients (data not shown). To calculate the incidence per general population, the incidence per patient was multiplied by the number of hospital discharges and normalised by the total population. This results in a higher incidence per general population for Germany – 592.1 (95% UI: 521.7–665.8) per 100,000 population – in comparison to the incidence per general population in the EU/EEA – 467.9 (95% UI: 456.2–480.2) per 100,000 population. This demonstrates that the number of hospital stays is an important factor, which needs to be considered, alongside other measures, in order to reduce the burden of HAIs per general population.

The results of the German and ECDC PPS 2016 indicate that the HAI prevalence may have slightly decreased in Germany since 2011 [25]. Nevertheless, the number of HAIs per 100,000 was still estimated to be higher in Germany – 735.6 (95% CI: 452.8–1,141.9) – than the EU/EEA estimate – 658.5 (95% CI: 437.0–957.6) [13,15].

The burden of UTIs and *C. difficile* was higher in Germany than the EU/EEA average, whereas the burden of HAP was lower in Germany (Figure 3, Supplementary Figure S5). This is in line with the ECDC PPS between 2011 and 2012, where Germany was among the countries with the highest proportions of UTIs and *C. difficile* infections among all HAIs within the PPS between 2011 and 2012. Since then, efforts have been made to enhance antibiotic stewardship and recommendations have been put in place in order to prevent catheter associated urinary tract infections [26]. The prevalence of UTIs was lower in the PPS 2016 and its proportion among all HAIs decreased, whereas prevalence of *C. difficile* infections in Germany increased [27]. Further research is needed to analyse whether these differences point towards more or less effective strategies to prevent these types of HAIs.

### Comparison of the BHAI and BCoDE methodologies

In principle, the BHAI methodology and the application of BCoDE for HAIs as in Cassini et al. [3] have the same approach: prevalence is converted to incidence per patient, which is then extrapolated to the population. Using Monte Carlo simulations, the number of HAIs is sampled and identical disease outcome trees are used to estimate the attributable burden of HAIs.

However, Cassini et al. used the median of LOI_PPS_ to estimate LOI for prevalence to incidence conversion, while BHAI uses a new method that was shown to be less prone to bias and performed more reliably in simulation studies [14].

Moreover, in our analyses we found that the stratified sampling approach in BCoDE [3,7], may overestimate the number of HAIs in strata with zero cases in the PPS, which became more frequent with smaller sample size in the range of those in the PPS conducted for individual countries. To address this, BHAI samples the number of HAIs in each simulation on the population level and then distributes cases to strata by an age and sex distribution. We carried out simulations using subsamples of the full ECDC PPS dataset between 2011 and 2012 to demonstrate that this methodological adjustment addressed the issue of overestimation in smaller samples (Methods, Supplementary Figure S7). Therefore, we recommend the use of BHAI in future efforts to estimate the health burden of HAIs in order to ensure comparability, particularly at the country level.

The new sampling approach in BHAI makes application to data collected with both the ECDC PPS patient-based (‘standard’) and unit-based (‘light’) protocols possible, whereas BCoDE can only be applied to the patient-based protocol. In contrast to the patient-based protocol, data for patients without HAIs are not collected with the unit-based protocol. Data on patients without HAIs are needed as denominator data for stratification in the original BCoDE approach, but not required for the BHAI methodology. Nevertheless, one limitation of the unit-based protocol is that the mean LA of patients is not recorded, which meant that we had to use hospital denominator data from the PPS. We therefore calculated LA from data of participating hospitals, based on the number of discharges and patient days from the previous year. However, in the patient-based protocol, the mean LA is recorded for each patient. In this case we suggest that LA and LOI are both calculated using the method by Willrich et al., since possible biases or errors in the estimation might cancel out in the division of LA by LOI during the conversion from prevalence to incidence [14].

### Limitations

The available evidence for transition probabilities for the outcome trees was derived from systematic reviews. Even though this may be the most transparent and unbiased approach to design outcome trees, it has to be noted that the underlying literature was sparse and of moderate to low quality [9]. With increasing evidence from the literature, outcome trees will improve and therefore should be reevaluated in the future. Transition probabilities in the outcome trees represent the likelihood of sequelae or death attributable to an infection. This justifies that the burden estimation is based on the number of HAIs, and not on the number of patients. Recurrent infections for each patient are implicitly reflected in the transition probabilities. By considering HAIs independently, we do not account for the fact that some patients have multiple HAIs. In addition, the probability of sequelae, recovery and death might not be independent. But in the German PPS sample, only 7% (28 patients with two or more HAIs/394 patients with one or more HAIs) of patients with a HAI had more than one HAI on the day of the PPS. Thus, the violation of the independence assumption likely results in minor overestimation.

We used the European McCabe score distribution for our estimation. The large number of acute care hospital beds in Germany might lead to avoidable hospital stays and a hospitalised patient population with less severe primary diseases than in other EU/EEA countries [22–24]. The European McCabe scores might therefore overestimate the severity of underlying diseases of German hospitalised patients. In turn, DALYs would be underestimated for Germany since the European McCabe distribution would assume lower remaining life expectancies.

Following the study of Cassini et al. we used hospital discharges of 2011 for all countries, although some countries conducted the PPS in 2012 [3]. In addition, LA was calculated using data from the previous year of the participating hospitals. This should not have significantly biased our results since the variation of LA and the number of discharges during the study period was low [13].

Another possible limitation of our study is the representativeness of the PPS data. Although hospital size was the only variable used for randomly sampling hospitals to obtain a representative sample of German hospitals, case mix and specialisation also influence HAI prevalence. For this reason, we also estimated the burden of HAIs from the larger convenience sample of German hospitals. This analysis led to higher estimates of the burden of HAIs, suggesting that a high uncertainty remains when estimating the burden of HAIs from PPS samples.

### Conclusion

In summary, the open-source BHAI R package, was applied to German and EU/EEA data to calculate the burden of HAIs at country level, and is applicable to other countries in or outside of the EU/EEA. In Germany, the burden of HAIs is higher than the burden of other communicable diseases. Despite the fact that Germany has a relatively low prevalence of HAIs compared with other European countries, the burden of HAIs in Germany is higher than the EU/EEA average [6]. A probable cause for the high burden of HAIs in Germany is the country’s large hospital patient population. It has been argued that the large numbers of acute care beds in Germany may lead to avoidable hospital stays [28]. Therefore, the reduction of avoidable hospital stays together with further focus on hygiene measures and infection prevention and control are important steps to reduce the burden of HAIs in Germany.

## Supplementary Data

1888000 Supplement_Figures-and-Tables

## Supplementary Data

650000 Supplement S1
