# Supplementary material for: Application of a new methodology and R package reveals a high burden of healthcare-associated infections (HAI) in Germany compared to the average in the European Union/European Economic Area, 2011 to 2012
Source: Euro Surveill. 2019 Nov 14;24(46):1900135. doi: 10.2807/1560-7917.ES.2019.24.46.1900135 (PMC6864977; doi:10.2807/1560-7917.ES.2019.24.46.1900135)
Supplement: Supplement_Figures-and-Tables [file 1900135_ZACHER_Supplement_Figures-and-Tables.pdf]

Application of a new methodology and R package reveals a high burden of healthcare-associated infections in Germany compared to the average in European Union/European Economic Area, 2011 to 2012

This supplementary material is hosted by *Eurosurveillance* as supporting information alongside the article ”Application of a new methodology and R package reveals a high burden of healthcare-associated infections in Germany compared to the average in European Union/European Economic Area, 2011 to 2012”, on behalf of the authors, who remain responsible for the accuracy and appropriateness of the content. The same standards for ethics, copyright, attributions and permissions as for the article apply. Supplements are not edited by *Eurosurveillance* and the journal is not responsible for the maintenance of any links or email addresses provided therein.

Supplementary Figures and Tables

Supplementary Figure S1: Estimation of the length of infection with different sample sizes (subsamples are taken from the ECDC PPS data for the EU/EEA) for five types of HAIs. Our methodology uses a weighted sum of the Grenander estimator and the mean using the sigmoid function (Willrich et al.[1]). The function is defined such that both estimators have weight 0.5 at a sample size of 500. With increasing sample size the Grenander estimator introduced by Willrich et al. gets more weight. We investigated the effect of the weighting by taking subsamples from LOI_{pps} (days from hospital admission until date of survey) from the ECDC PPS and calculated LOI estimates using 1000 bootstraps.

[1] Willrich N, Haller S, Eckmanns T, Zacher B, Kärki T, Plachouras D, et al. From prevalence to incidence - a new approach in the nosocomial setting. bioRxiv. 2019:554725.

Supplementary Figure S1

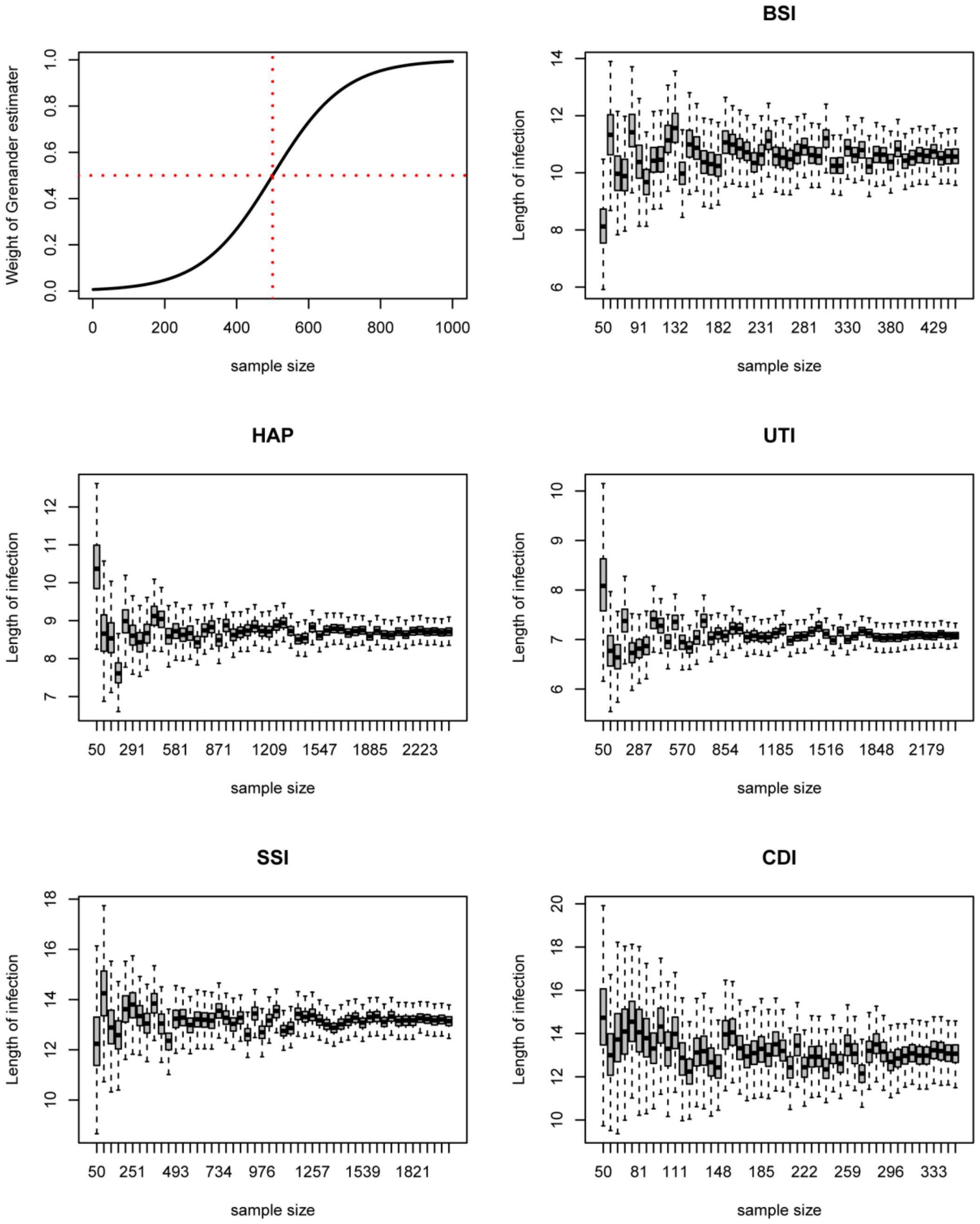

Bars show the median and 95% uncertainty intervals.

LOI, length of infection; HAI, healthcare-associated infection; PPS, point prevalence survey; LOI, length of infection; HAP, healthcare-associated pneumonia; UTI, healthcare-associated urinary tract infection; SSI, surgical site infection; CDI, healthcare-associated *Clostridium difficile* infection); BSI, healthcare-associated primary bloodstream infection; EU/EEA, European Union/European Economic Area; ECDC, European Center for Disease Prevention and Control

Supplementary Figure S2: Estimated length of infection of the five types of HAIs for 1,000 Monte Carlo simulations for the German PPS. The median and 95% uncertainty intervals are shown on the x-axis. Germany, 2011

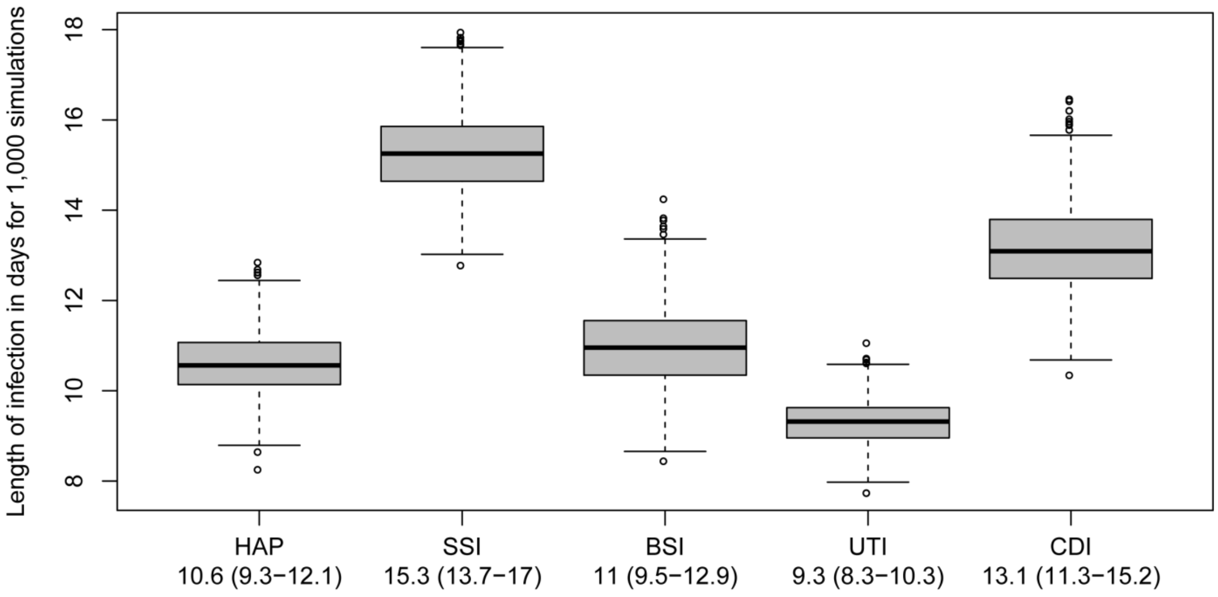

Bars show the median and 95% uncertainty intervals.

PPS, point prevalence survey; HAP, healthcare-associated pneumonia; UTI, healthcare-associated urinary tract infection; SSI, surgical site infection; CDI, healthcare-associated *Clostridium difficile* infection); BSI, healthcare-associated primary bloodstream infection

Supplementary Figure S3: Annual burden of five types of HAIs (HAP, BSI, UTI, SSI, CDI) stratified by age and gender for the German convenience sample. (A) DALYs per 100,000 in each stratum. (B) Total number of DALYs in each stratum. Germany, 2011

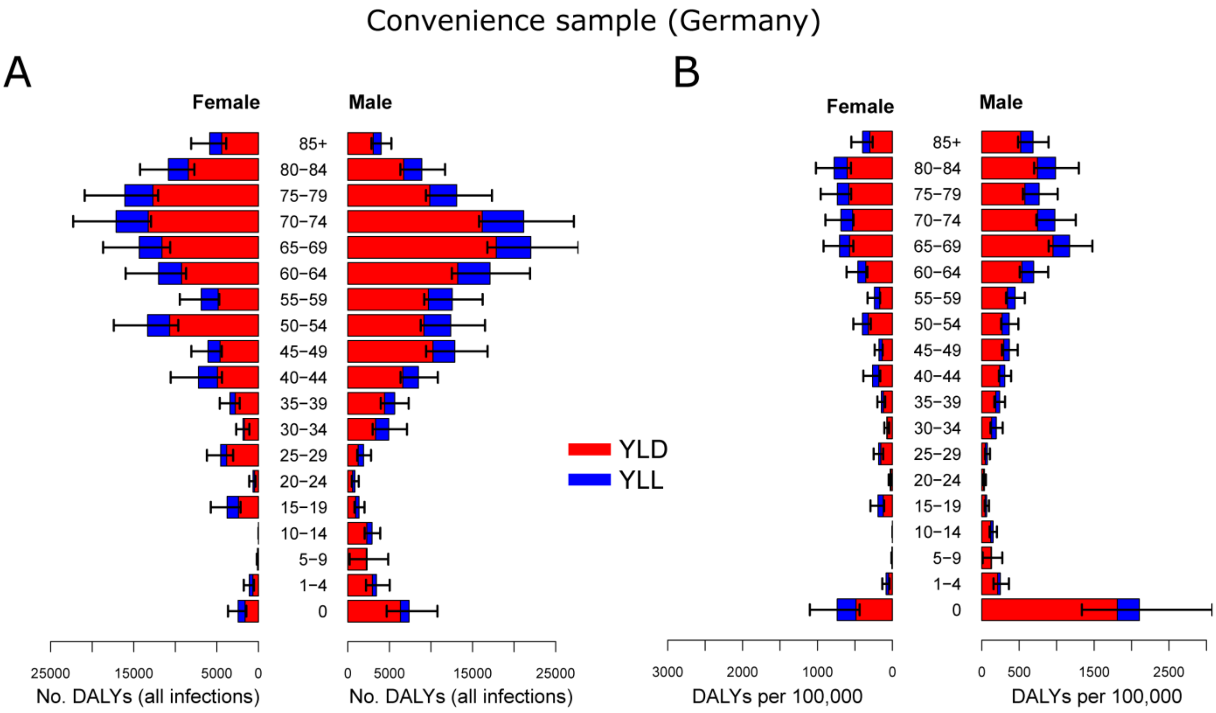

Bars show the median and 95% uncertainty intervals.

DALY, disability-adjusted life year; HAI, healthcare-associated infection; HAP, healthcare-associated pneumonia; UTI, healthcare-associated urinary tract infection; SSI, surgical site infection; CDI, healthcare-associated *Clostridium difficile* infection); BSI, healthcare-associated primary bloodstream infection

Supplementary Figure S4: Annual number of HAIs plotted against the annual number of attributable deaths for five types of HAIs in Germany (German convenience sample). The width of the bubbles is proportional to the number of DALYs per year. Germany, 2011

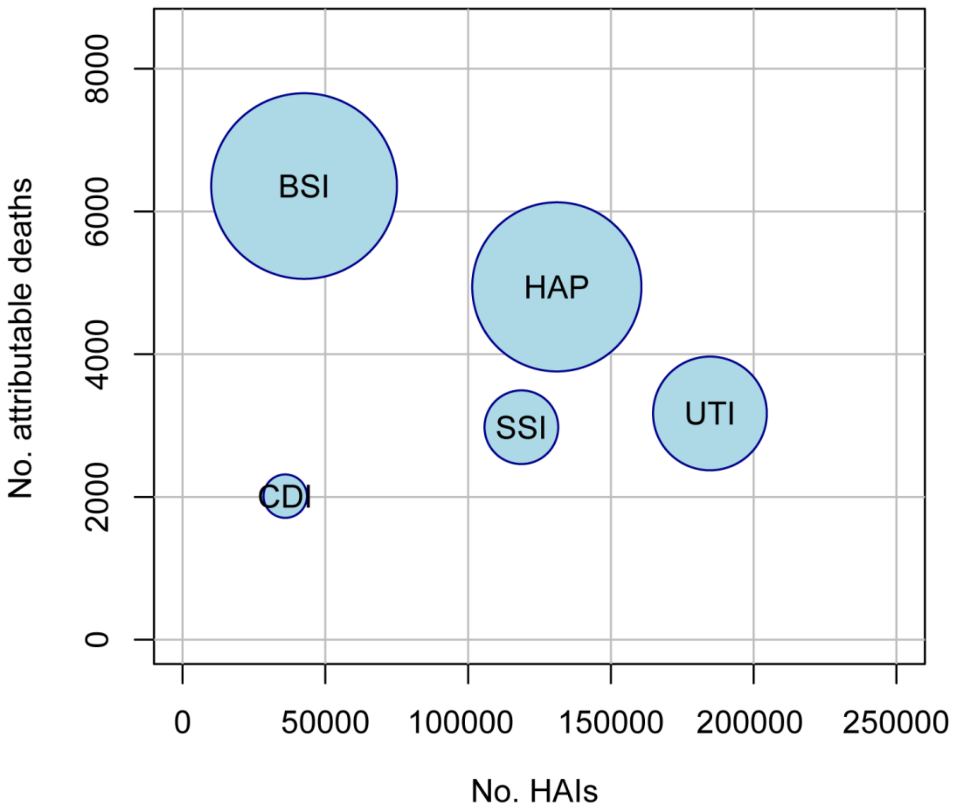

HAI, healthcare-associated infection; HAP, healthcare-associated pneumonia; UTI, healthcare-associated urinary tract infection; SSI, surgical site infection; CDI, healthcare-associated *Clostridium difficile* infection); BSI, healthcare-associated primary bloodstream infection

Supplementary Figure S5: Annual burden of five types of HAIs in Germany and the EU/EEA. (A) HAIs, (B) attributable deaths, (C) DALYs per 100,000 population.

Bars show the median and 95% uncertainty intervals. Germany, EU/EEA 2011-2012

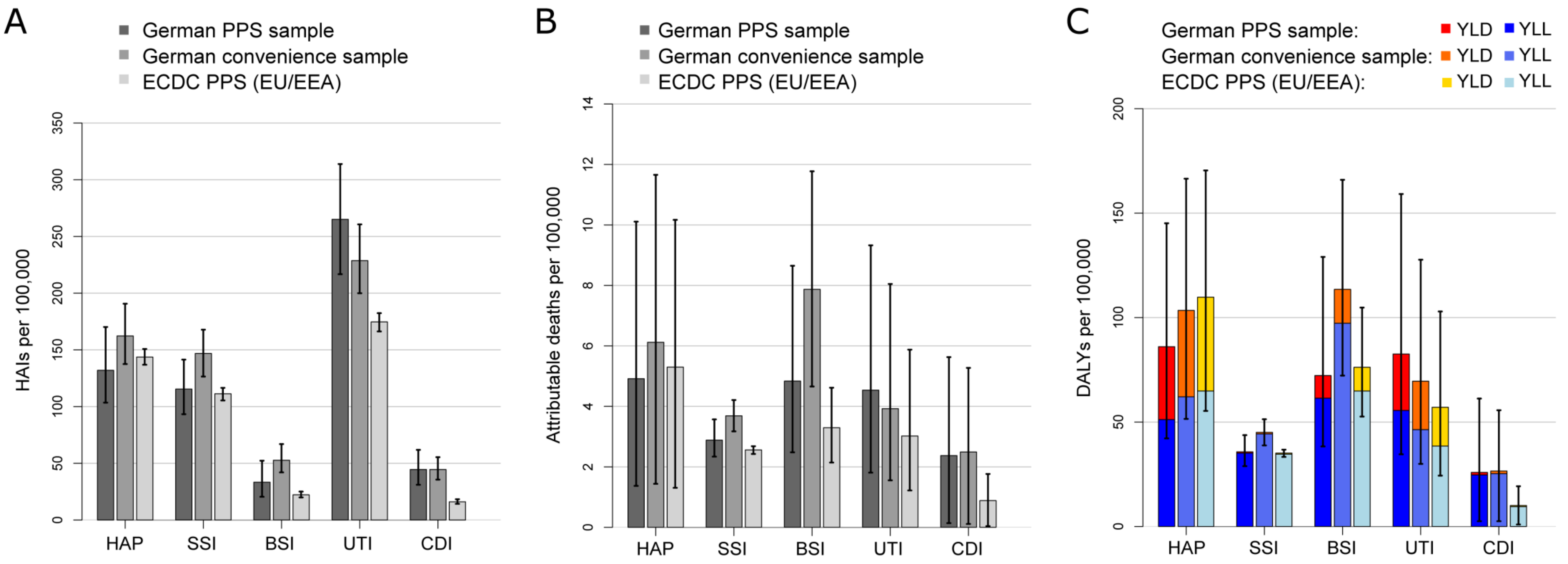

Bars show the median and 95% uncertainty intervals.

EU/EEA, European Union/European Economic Area; HAI, healthcare associated infection; DALY, disability-adjusted life year; YLL, years of life lost; YLD, years lived with disability; HAI, healthcare-associated infection; HAP, healthcare-associated pneumonia; UTI, healthcare-associated urinary tract infection; SSI, surgical site infection; CDI, healthcare-associated *Clostridium difficile* infection); BSI, healthcare-associated primary bloodstream infection

Supplementary Figure S6: Ratios of the German and the EU/EEA burden of HAIs are calculated for all age groups. (A-C) Ratio between the German PPS sample and ECDC PPS sample. (D-E) Ratio between the German convenience sample and ECDC PPS sample. Germany, EU/EEA 2011-2012

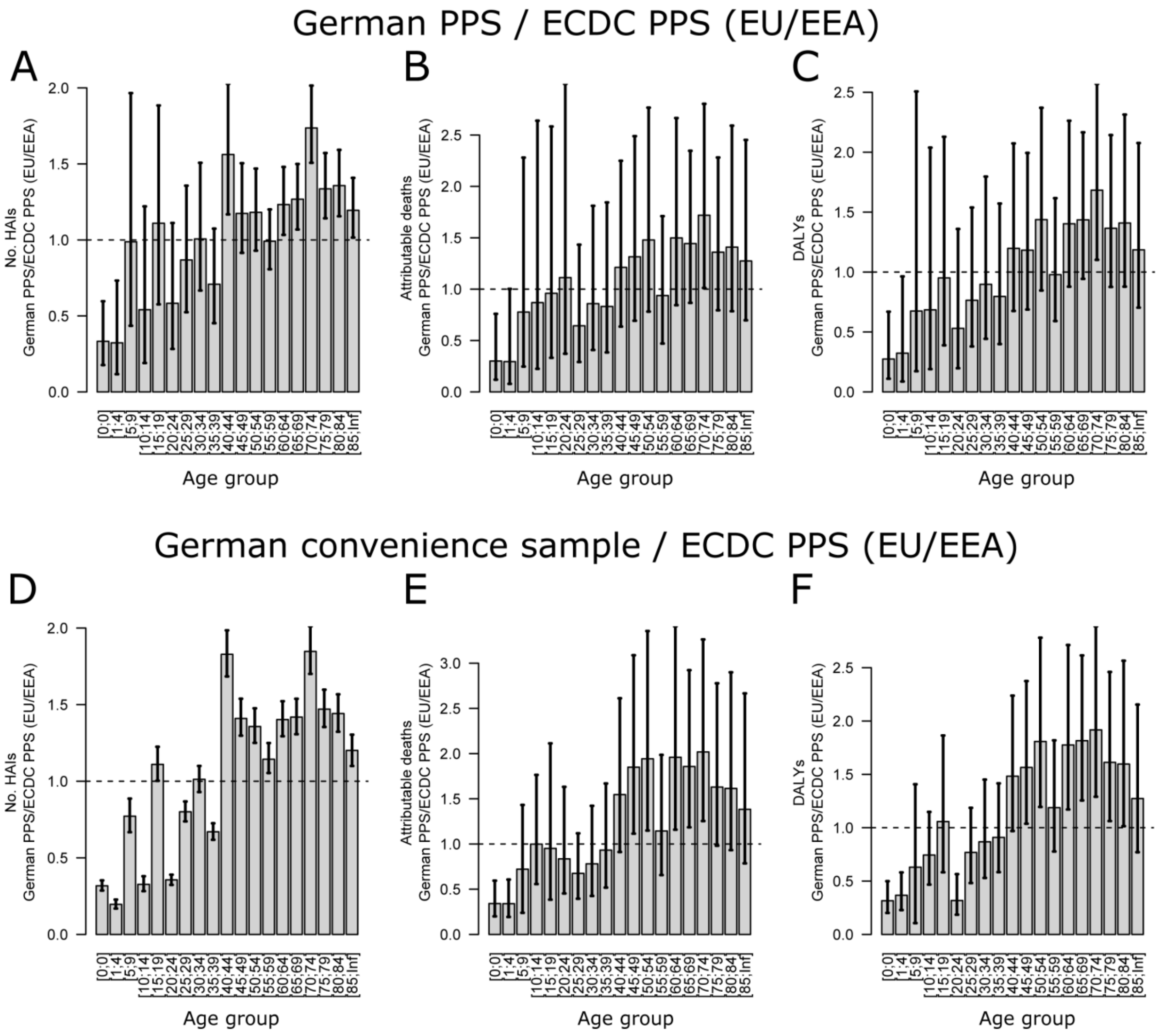

Bars show the median and 95% uncertainty intervals.

DALY, disability-adjusted life year; HAI, healthcare-associated infection; PPS, point prevalence survey; EU/EEA, European Union/European Economic Area; ECDC, European Center for Disease Prevention and Control

Supplementary Figure S7: Comparison of different sampling strategies with simulated PPS data (based on parameters from ECDC PPS). Estimated DALYs (left) and number of yearly cases (right) for healthcare-associated pneumonia (HAP) are shown. PPS data are simulated for different sample sizes. Top: Stratified sampling as proposed by Cassini et al.[2] is carried out showing an overestimation for small samples. Middle: BHAI R package gives stable results over a wide range of sample sizes. Bottom: Application of a prior distribution for age and gender (obtained from the full European sample) stabilizes DALY estimation with small samples. The golden dashed line shows the BHAI R-package estimate of the full European sample.

[2] Cassini A, Plachouras D, Eckmanns T, Abu Sin M, Blank HP, Ducomble T, et al. Burden of Six Healthcare-Associated Infections on European Population Health: Estimating Incidence-Based Disability-Adjusted Life Years through a Population Prevalence-Based Modelling Study. PLoS medicine. 2016;13(10):e1002150

Supplementary Figure S7

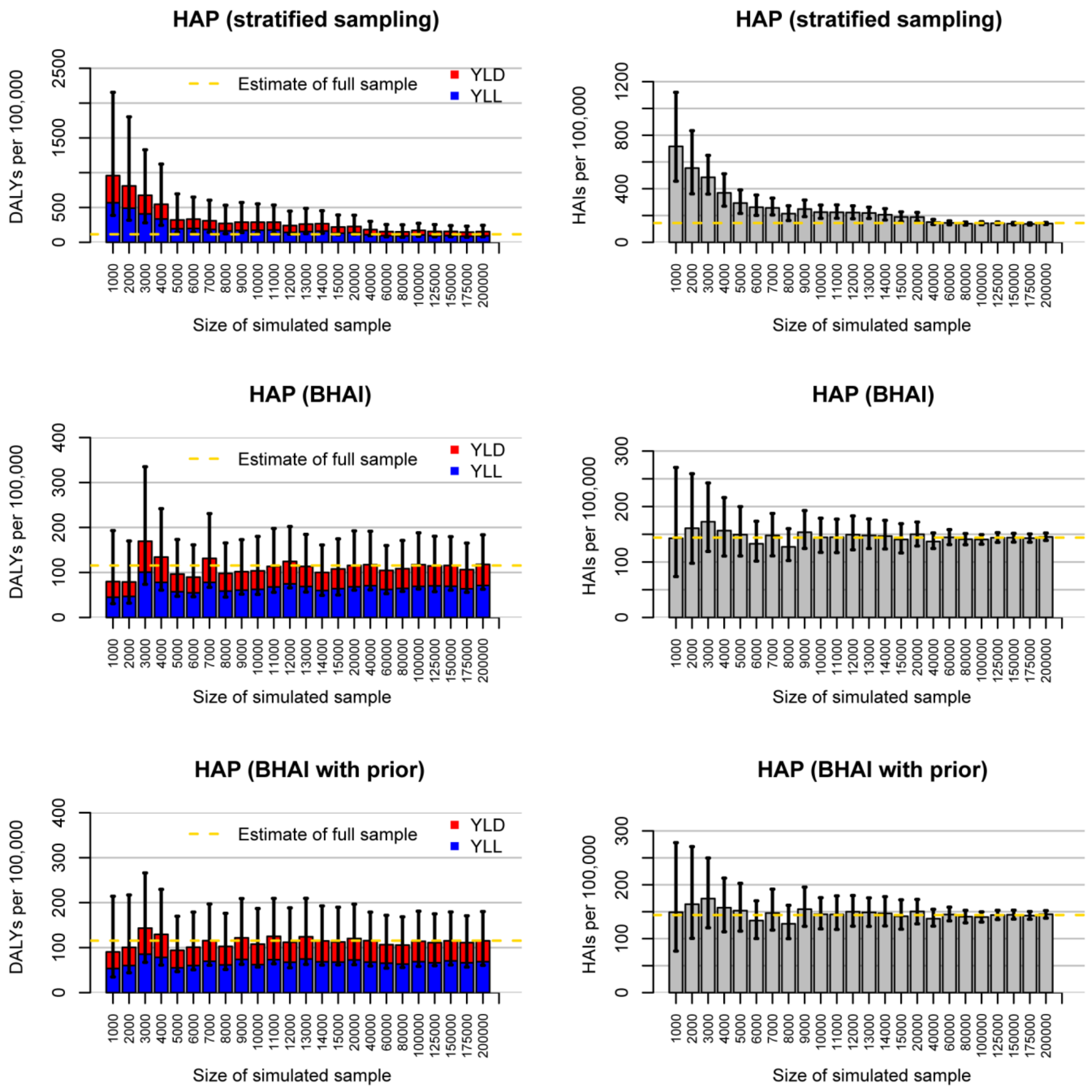

Bars show the median and 95% uncertainty intervals.

PPS, point prevalence survey; DALY, disability-adjusted life year; YLL, years of life lost; YLD, years lived with disability; HAP, healthcare-associated pneumonia; ECDC, European Center for Disease Prevention and Control

Supplementary Table S1: Annual burden of five types of HAIs is shown for the German convenience sample. Estimation was carried out using the BHAI methodology and R package. Point estimates (median) and 95% uncertainty intervals are shown. Germany, 2011

Type of HAI	Sample	No. HAIs	No. attribuatable deaths	No. DALYs	No. YLLs	No. YLDs
**HAP**	convenience	131,072 (111,049 - 154,044)	4,944 (1,161 - 9,416)	83,522 (41,589 - 134,475)	50,123 (11,769 - 95,467)	33,421 (20,783 - 48,731)
**SSI**	convenience	118,656 (102,184 - 135,540)	2,977 (2,564 - 3,401)	36,371 (31,335 - 41,439)	35,787 (30,820 - 40,880)	575 (466 - 695)
**BSI**	convenience	42,554 (33,919 - 54,063)	6,356 (3,761 - 9,509)	91,677 (58,334 - 134,050)	78,555 (46,484 - 117,528)	13,521 (7,593 - 21,154)
**UTI**	convenience	184,705 (161,535 - 210,541)	3,170 (1,256 - 6,501)	56,153 (24,190 - 103,148)	37,432 (14,825 - 76,753)	17,563 (6,925 - 33,462)
**CDI**	convenience	35,958 (28,786 - 44,748)	2,011 (89 - 4,260)	21,427 (2,020 - 44,940)	20,418 (899 - 43,243)	942 (175 - 1,988)
**ALL**	convenience	513,729 (473,840 - 556,654)	19,672 (13,921 - 26,310)	290,228 (215,305 - 372,734)	224,939 (160,973 - 298,122)	65,988 (46,856 - 89,194)

DALY, disability-adjusted life year; YLL, years of life lost; YLD, years lived with disability; HAI, healthcare-associated infection; HAP, healthcare-associated pneumonia; UTI, healthcare-associated urinary tract infection; SSI, surgical site infection; CDI, healthcare-associated *Clostridium difficile* infection); BSI, healthcare-associated primary bloodstream infection

Supplementary Table S2: Comparison of the number of DALYs of five types of HAIs (German convenience sample) with other communicable diseases in Germany[3]. Point estimates (median) and 95% uncertainty intervals are shown. Germany, 2005-2007 and 2011

Infection	Sample / Source	DALYs
BSI	convenience	91,677 (58,334 - 134,050)
HAP	convenience	83,522 (41,589 - 134,475)
**HAP**	German PPS	69,508 (34,042 - 117,232)
**UTI**	German PPS	66,701 (27,890 - 128,543)
**BSI**	German PPS	58,350 (30,940 - 104,227)
UTI	convenience	56,153 (24,190 - 103,148)
SSI	convenience	36,371 (31,335 - 41,439)
**Influenza**	Plass et al. (2013)	33,116 (29,504 – 36,849)
**SSI**	German PPS	28,842 (23,313 - 35,303)
CDI	convenience	21,427 (2,020 - 44,940)
**CDI**	German PPS	20,890 (2,023 - 49,443)
***Salmonella* spp.**	Plass et al. (2013)	19,115 (14,803 – 24,328)
**Hepatitis B virus**	Plass et al. (2013)	8,708 (7,335 – 10,163)
**Measles virus**	Plass et al. (2013)	704 (413 – 1,066)

PPS, point prevalence survey; DALY, disability-adjusted life year; HAP, healthcare-associated pneumonia; UTI, healthcare-associated urinary tract infection; SSI, surgical site infection; CDI, healthcare-associated *Clostridium difficile* infection); BSI, healthcare-associated primary bloodstream infection

[3] Plass D, Mangen MJ, Kraemer A, Pinheiro P, Gilsdorf A, Krause G, et al. The disease burden of hepatitis B, influenza, measles and salmonellosis in Germany: first results of the burden of communicable diseases in Europe study. Epidemiology and infection. 2014;142(10):2024-35

Supplementary Table S3: Annual burden of five types of HAIs is shown for the full ECDC PPS sample (EU/EEA, 2011-2012). Estimation was carried out using the BHAI methodology and R package. Point estimates (median) and 95% uncertainty intervals are shown. Germany, 2011

Type of HAI	No. HAIs	No. attribuatable deaths	No. DALYs	No. YLLs	No. YLDs
**HAP**	112,314 (100,874 - 126,737)	16,665 (10,826 - 23,342)	385,220 (265,927 - 529,755)	327,945 (213,049 - 459,333)	56,804 (33,277 - 87,774)
**SSI**	726,684 (692,019 - 762,393)	26,782 (6,600 - 51,402)	554,838 (279,607 - 861,677)	327,956 (80,819 - 629,441)	220,048 (137,929 - 305,658)
**BSI**	883,161 (840,632 - 922,074)	15,277 (6,162 - 29,708)	288,472 (123,028 - 520,265)	194,594 (78,489 - 378,414)	86,810 (34,288 - 167,955)
**UTI**	562,723 (532,871 - 589,216)	12,950 (12,263 - 13,559)	177,459 (168,189 - 185,799)	174,809 (165,536 - 183,039)	2,749 (2,307 - 3,142)
**CDI**	81,124 (72,010 - 92,585)	4,465 (195 - 8,922)	50,575 (4,657 - 97,290)	47,918 (2,090 - 95,754)	2,165 (445 - 4,490)
**ALL**	2,365,466 (2,306,018 - 2,427,774)	77,483 (51,502 - 106,982)	1,465,822 (1,086,252 - 1,905,451)	1,089,473 (738,862 - 1,456,882)	374,074 (263,147 - 485,457)

DALY, disability-adjusted life year; YLL, years of life lost; YLD, years lived with disability; HAI, healthcare-associated infection; HAP, healthcare-associated pneumonia; UTI, healthcare-associated urinary tract infection; SSI, surgical site infection; CDI, healthcare-associated *Clostridium difficile* infection); BSI, healthcare-associated primary bloodstream infection

Supplementary Table S4: Comparison of the number of DALYs of five types of HAIs with other communicable diseases in Germany estimated in Global Burden of Disease 2010[4]. Point estimates and 95% uncertainty/confidence intervals are shown. Germany, 2010-2011

Rank	Cause	No. DALYs
1	Cardiovascular and circulatory diseases	4,623,567
2	Cancer	4,260,565
3	Musculoskeletal disease	3,771,654
4	Mental and behavioral disorders	2,765,881
5	Diabetes, urogenital, blood-related and endocrine disorders	1,470,391
6	Neurological disorders	1,156,588
7	Other non-communicable diseases	1,153,661
8	Unintentional injury	1,074,631
9	Chronic respiratory diseases	1,068,544
10	Transport injuries	492,386
11	Diarrhea, lower respiratory tract disease, and other infectious diseases	489,841
12	Intentional injuries	435,432
13	Cirrhosis	431,304
14	Digestive diseases	390,476
**15**	**Healthcare-associated infections HAP, BSI, UTI, SSI, CDI (German PPS)**	**248,920 (178,693 - 336,239)**
16	Neonatal disorders	168,110
17	Nutritional deficiencies	68,860
18	Other communicable diseases	45,519
19	HIV/AIDS and tuberculosis	44,141
20	NTDs and malaria	11,022
21	Maternal disorders	5,558
22	War and desasters	0

DALY, disability-adjusted life year; HAP, healthcare-associated pneumonia; UTI, healthcare-associated urinary tract infection; SSI, surgical site infection; CDI, healthcare-associated *Clostridium difficile* infection); BSI, healthcare-associated primary bloodstream infection

[4] Murray CJ, Ezzati M, Flaxman AD, Lim S, Lozano R, Michaud C, et al. GBD 2010: a multi-investigator collaboration for global comparative descriptive epidemiology. Lancet (London, England). 2012;380(9859):2055-8
Plass D, Vos T, Hornberg C, Scheidt-Nave C, Zeeb H, Kramer A. Trends in disease burden in Germany: results, implications and limitations of the Global Burden of Disease study. Deutsches Arzteblatt international. 2014;111(38):629-38
