# Supplementary material for: Application of a new methodology and R package reveals a high burden of healthcare-associated infections (HAI) in Germany compared to the average in the European Union/European Economic Area, 2011 to 2012
Source: Euro Surveill. 2019 Nov 14;24(46):1900135. doi: 10.2807/1560-7917.ES.2019.24.46.1900135 (PMC6864977; doi:10.2807/1560-7917.ES.2019.24.46.1900135)
Supplement: Supplement S1 [file 1900135_ZACHER_Script.zip › Eurosurveillance_Disclaimer.pdf]

This supplementary material is hosted by *Eurosurveillance* as supporting information alongside the article ”Application of a new methodology and R package reveals a high burden of healthcare-associated infections in Germany compared to the average in European Union/European Economic Area, 2011 to 2012”, on behalf of the authors, who remain responsible for the accuracy and appropriateness of the content. The same standards for ethics, copyright, attributions and permissions as for the article apply. Supplements are not edited by *Eurosurveillance* and the journal is not responsible for the maintenance of any links or email addresses provided therein.
