# Supplementary material for: Application of a new methodology and R package reveals a high burden of healthcare-associated infections (HAI) in Germany compared to the average in the European Union/European Economic Area, 2011 to 2012
Source: Euro Surveill. 2019 Nov 14;24(46):1900135. doi: 10.2807/1560-7917.ES.2019.24.46.1900135 (PMC6864977; doi:10.2807/1560-7917.ES.2019.24.46.1900135)
Supplement: Supplement S1 [file 1900135_ZACHER_Script.zip › BHAI.zip!/BHAI/BHAI/vignettes/BHAI.pdf]

Estimation of the burden of healthcare-associated infections from point prevalence surveys

Benedikt Zacher, Sebastian Haller, Niklas Willrich, Jan Walter,
Alessandro Cassini, Diamantis Plachouras, Carl Suetens,
Michael Behnke, Petra Gastmeier, Lothar H. Wieler, Tim Eckmanns

August 13, 2019

Contents

1 Introduction **2**

2 The workflow of *BHAI* step-by-step **2**
2.1 Step 1: Prevalence sampling 3
2.2 Step 2: Prevalence to incidence conversion 3
2.3 Step 3: Extrapolation to population level 4
2.4 Step 4: Redistiribution of cases in age and gender strata 4
2.5 Step 5: Adjusting for comorbidities 6
2.6 Creating a PPS data object 7

3 Application of *BHAI* **8**
3.1 Summary tables 8
3.2 Summary plots 8
3.3 Comparison of multiple results 10

4 Stratified vs. unstratified sampling **12**

5 Sample size and robustness of the estimates **15**

6 Concluding Remarks **16**

1 Introduction

The estimation of the burden of healthcare-associated infections (HAIs) is an ongoing challenge. The *BHAI* package implements an approach inspired by the methodology of the Burden of Communicable Diseases in Europe (BCoDE) [2, 1] to estimate the *b*urden of *h*ealthcare-*a*ssociated *i*nfections (*BHAI*) from point prevalence surveys (PPS). The modified methodology provides estimates which are stable also for small sample sizes which enables a robust burden estimation in individual countries. *BHAI* estimates the burden of HAIs in disability-adjusted life years (DALYs), number of infections as well as number of deaths per year.
We demonstrate the use of *BHAI* by estimating the burden of five common HAIs from German and European PPS data. These include healthcare-associated pneumonia (HAP), healthcare-associated urinary tract infection (UTI), surgical site infection (SSI), healthcare-associated Clostridium difficile infection (CDI), and healthcare-associated primary bloodstream infection (BSI). In order to load the package please type:

```
library(BHAI)
```

2 The workflow of *BHAI* step-by-step

We will now explain the workflow of *BHAI* at the example of the German PPS data from 2011. A graphical overview of all steps is illustrated in Figure 1. In short *BHAI* performs a predefined number (default: 1,000) of Monte Carlo simulations of the following steps:

1. Prevalence is sampled for each HAI using observed number of cases and suvery patients.
2. Prevalence is converted to incidence per patient using a modified Rhame-Sudderth formula.
3. Incidence per patient is multiplied with the number of hospital discharges to get the overall number of cases per year.
4. Accoring to a predefined age and gender distribution (obtained from the PPS) for each HAI, cases are distributed into respective age and gender strata.
5. Disease outcome trees are simulated to estimate the number of deaths and DALYs in each stratum. *BHAI* adjusts for comorbidities using a predefined McCabe score distribution.

Before starting, we load `german_pps_2011_repr` which includes preprocessed data from the German PPS 2011, information about the German population and the number of hosptial discharges. We start with the representative sample which consists of 46 hospital and 9,626.

```
data(german_pps_2011_repr)
objects()
##  [1] "hospital_discharges"
##  [2] "length_of_stay"
##  [3] "loi_pps"
##  [4] "mccabe_life_exp"
##  [5] "mccabe_scores_distr"
##  [6] "num_hai_patients"
##  [7] "num_hai_patients_by_stratum"
##  [8] "num_hai_patients_by_stratum_prior"
##  [9] "num_survey_patients"
## [10] "population"
```

In the following sections, we will describe the data and explain how it is used in the different computations of *BHAI*.

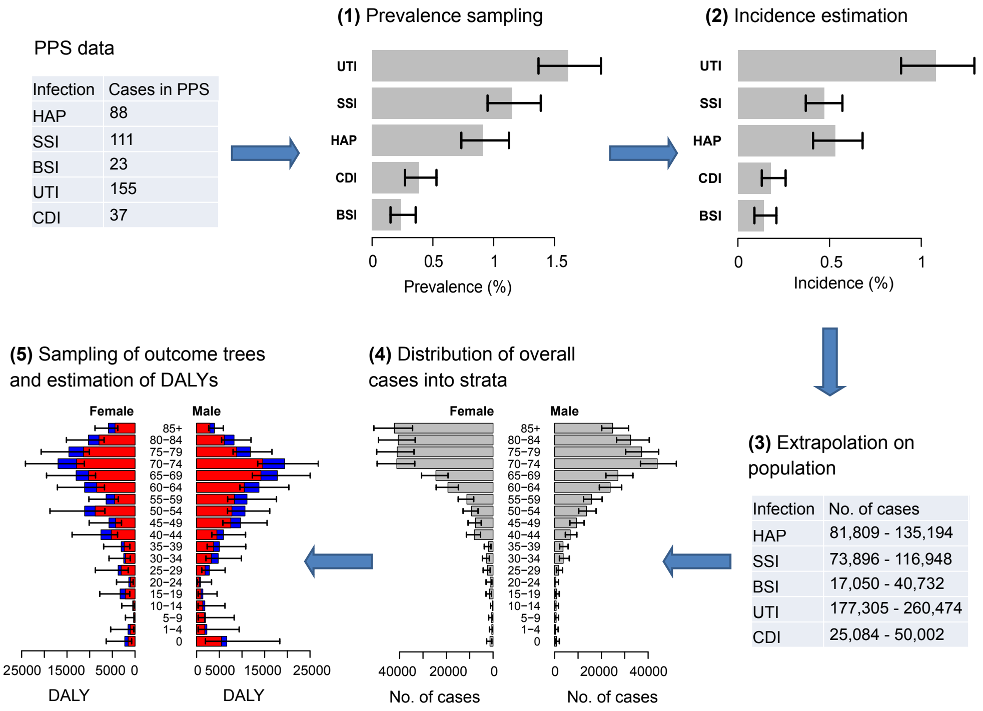

Figure 1: Workflow of *BHAI*

2.1 Step 1: Prevalence sampling

BHAI calculates prevalence for each HAI using the observed number of cases (`num_hai_patients`) and the total number of patients (`num_survey_patients`) in the PPS:

```
num_hai_patients
## HAP SSI BSI UTI CDI
##  88 111  23 155  37
num_survey_patients
## [1] 9626
```

The uncertainty about the prevalence is incorporated by sampling from a mixture of two beta distributions, which form the exact mid-P-Clopper-Pearson confidence interval. The use of this mixture distribution gives uncertainty intervals (2,5% and 97,5% quantiles) that accurately reflect the exact confidence intervals. Note that sampling from this mixture distirbution is very similar to a Bayesian posterior sampling using an uninformative Jeffery's prior.

2.2 Step 2: Prevalence to incidence conversion

In the next step, a modified Rhame-Sudderth formula is applied to convert prevalence to incidence (per patient): $Incidence = Prevalence * \frac{length\_of\_stay}{length\_of\_infection}$ [3]. The average length of stay (`length_of_stay`) of all patients in the survey was computed from denominator data of participating hospitals.

```
length_of_stay
## [1] 6.181485
```

One crucial question when applying the Rhame-Sudderth formula is how to estimate the length of infection (LOI) from PPS data. The Rhame-Sudderth formula defines the length of infections as the time of infection onset until the date of hospital discharge. However a PPS usually only collects the time from infection onset until the date of survey (LOI_{PPS}). Estimators of LOI that have been used in the past are the median or mean of LN-INT which are ad-hoc approximations for the 'true' length of infection. A more theoretically justified method was recently developed by [3], but a large number of observations is needed for its application. Thus this approach is not applicable directly to the german representative sample. However Willrich et al. [3] showed that the mean - which is biased but more robust with small samples - performed well in most applications. *BHAI* therefore uses a weighted sum of both estimators using the logistic function. The function is defined such that both estimators have weight 0.5 at a sample size of 500. Thus, with increasing sample size the estimator by [3] gets more weight and with small sample sizes the mean gets more weight. *BHAI* incorporates uncertainty about the LOI estimate using bootstraps in each simulation.
We use LOI_{PPS} form the convenience sample in order to reduce uncertainty and improve estimates for burden estimation of the german representative sample, with the mean to estimate the length of infection, which is stored in `loi_pps` stores.

```
str(loi_pps)
## List of 5
##  $ HAP: num [1:408] 29 12 12 5 13 7 6 33 2 1 ...
##  $ SSI: num [1:539] 34 24 26 2 27 6 22 1 6 29 ...
##  $ BSI: num [1:142] 15 5 7 13 8 37 6 8 7 22 ...
##  $ UTI: num [1:516] 7 10 2 5 8 10 5 3 9 4 ...
##  $ CDI: num [1:142] 18 7 22 10 15 26 12 3 20 6 ...
```

2.3 Step 3: Extrapolation to population level

BHAI extrapolates the incidence per patient to the whole population by multiplication with the number of hospital discharges. The population data and number of hospital discharges were obtained from the Eurostat database (http://ec.europa.eu/eurostat/) and are stored in:

```
hospital_discharges
## [1] 19868533
population
## [1] 80767463
```

2.4 Step 4: Redistiribution of cases in age and gender strata

After having estimated in total number of yearly cases in steps 1-3 *BHAI* continues with calculating disablility-adjusted life years (DALYs). In order to do this, cases need to be distributed to age and gender categories to account for different remaining life expectancies. This distribution is obtained from the PPS and stored in, `num_hai_patients_by_stratum` which contains the number of cases in each stratum for all HAIs:

```
names(num_hai_patients_by_stratum)
## [1] "HAP" "SSI" "BSI" "UTI" "CDI"
num_hai_patients_by_stratum$HAP
##          F  M
## [0;0]    0  0
## [1;4]    0  0
```

```
## [5;9]    0  0
## [10;14]  0  0
## [15;19]  0  0
## [20;24]  0  0
## [25;29]  0  1
## [30;34]  0  0
## [35;39]  0  2
## [40;44]  1  0
## [45;49]  0  0
## [50;54]  2  4
## [55;59]  3  6
## [60;64]  1  5
## [65;69]  2  6
## [70;74]  6 12
## [75;79]  5  7
## [80;84]  5 10
## [85;Inf] 4  6
```

The stratified data has many empty strata (zeros) even for HAP which is one of the more prevalent infections. Using this data to redistirbute cases will result in an unrealistic distribution of DALYs probably leading to an overestimation in younger age group (because of the zeros) and an overestimation in older age groups (because all the probability mass lies there).
One way to deal with small sample sizes is to use Bayesian analysis to include prior knowledge about the distribution. A prior would tell us that - although we observed zero cases - the probability that there are no cases in a stratum is unlikely. Bayesian analysis combines this prior information with the evidence from the observed data in a posterior distribution of the 'true' parameters (i.e. relative frequencies in all strata). As a consequence, the posterior probability will be shifted away from the zero. In this scenario the posterior is a multinomial likelihood with a Dirichlet prior, which is parameterized by prior weights. Here we simply use the number of cases from the larger german convenience sample as prior weights:

```
num_hai_patients_by_stratum_prior$HAP
##           F  M
## [0;0]     2  0
## [1;4]     1  0
## [5;9]     2  0
## [10;14]   0  1
## [15;19]   3  1
## [20;24]   0  1
## [25;29]   0  2
## [30;34]   0  3
## [35;39]   1  4
## [40;44]   6  5
## [45;49]   3  7
## [50;54]   5 19
## [55;59]  13 16
## [60;64]  12 23
## [65;69]  11 27
## [70;74]  26 49
## [75;79]  25 51
## [80;84]  26 33
## [85;Inf] 22 21
```

Note that this distribution also contains empty strata - although not as many as the representative sample. Since all prior weights have to be > 0 *BHAI* adds a small pseudocount: $0.001 * sum(prior\_weights)$. In each Monte Carlo simulation a sample is drwan from the posterior which incorporates uncertainty about the age and gender distribution. This distribution is then multiplied with the overall number of yearly cases to get the stratified number of cases for each HAI.

2.5 Step 5: Adjusting for comorbidities

The remaining life expectancy is not only affected by the age of patients but also by the severity of the underlying disease. *BHAI* adjusts for this comorbidity using the McCabe score as described in [1]. Patients are grouped into three classes based on whether the underlying disease is non fatal (normal life expectancy), ultimately fatal (average expected life expectancy of 3 years) or rapidly fatal (average expected life expectancy of 0.5 years). This information is stored as a list of matrices in `mccabe_life_exp`.

```
names(mccabe_life_exp)
## [1] "NONFATAL" "ULTFATAL" "RAPFATAL"
mccabe_life_exp$NONFATAL
##           Female  Male
## [0;0]      85.68 85.68
## [1;4]      83.63 83.63
## [5;9]      78.76 78.76
## [10;14]    73.79 73.79
## [15;19]    68.83 68.83
## [20;24]    63.88 63.88
## [25;29]    58.94 58.94
## [30;34]    54.00 54.00
## [35;39]    49.09 49.09
## [40;44]    44.23 44.23
## [45;49]    39.43 39.43
## [50;54]    34.72 34.72
## [55;59]    30.10 30.10
## [60;64]    25.55 25.55
## [65;69]    21.12 21.12
## [70;74]    16.78 16.78
## [75;79]    12.85 12.85
## [80;84]     9.34  9.34
## [85;Inf]    5.05  5.05
```

To apply the correction for comorbidity, *BHAI* has to distribute patients in McCabe categories, i.e. the McCabe score distribution for each HAI, age and gender is needed. The german data was collected using a 'light protocol', which did not record the McCabe scores. Thus we assume the McCabe score distribution of the european PPS (i.e. cumulative data from all countries that recored McCabe score with the 'standard protocol') for Germany. `mccabe_scores_distr` is a list containing for all HAIs, age, gender strata and McCabe categories the number of cases in the PPS:

```
 # HAIs
names(mccabe_scores_distr)
## [1] "BSI" "HAP" "UTI" "SSI" "CDI"
# McCabe categories
names(mccabe_scores_distr$HAP)
## [1] "NONFATAL" "RAPFATAL" "ULTFATAL"
```

```
# Observed non fatal HAP cases (from EU sample)
mccabe_scores_distr$HAP$NONFATAL
##           F  M
## [0;0]    14 28
## [1;4]     8 10
## [5;9]     0  3
## [10;14]   4  3
## [15;19]   6  8
## [20;24]   1 13
## [25;29]   3 17
## [30;34]   8 11
## [35;39]   2 22
## [40;44]  12 25
## [45;49]  20 21
## [50;54]  19 34
## [55;59]  21 47
## [60;64]  22 56
## [65;69]  29 57
## [70;74]  50 86
## [75;79]  66 78
## [80;84]  47 71
## [85;Inf] 62 55
```

BHAI computes a probability distribution of McCabe scores for each HAI, age and gender stratum and distributes cases by multiplication with the number of estimated cases in each simulation. Finally disease outcome trees are simulated - i.e. disabilty weights, durations and transition probabilities are sampled - and DALYs are calculated with respect to the remaining life expectancy in each stratum.

2.6 Creating a PPS data object

Now we have all the data ready for application. For reasons of clarity and comprehensibility *BHAI* summarizes this heterogeneous data in a *PPS* object. We use the `PPS` function to summarize the data of the german representative PPS sample in `german_pps_repr`:

```
german_pps_repr =
  PPS(num_hai_patients = num_hai_patients,
      num_hai_patients_by_stratum = num_hai_patients_by_stratum,
      num_hai_patients_by_stratum_prior = num_hai_patients_by_stratum_prior,
      num_survey_patients = num_survey_patients,
      length_of_stay = length_of_stay,
      loi_pps = loi_pps,
      mccabe_scores_distr = mccabe_scores_distr,
      mccabe_life_exp = mccabe_life_exp,
      hospital_discharges = hospital_discharges,
      population = population,
      country="Germany (representative sample)")
```

Upon construction of the *PPS* object an extensive number of data checks are executed to ensure the validity of the data. This is to make sure that the data is consistent and valid before application. Typing the name of the PPS object will show a very basic summary of the PPS object:

```
german_pps_repr
##   Country: Germany (representative sample)
##   Patients in PPS: 9,626
##   Patients with HAI: 414
##   HAIs: HAP, SSI, BSI, UTI, CDI
```

3 Application of *BHAI*

Now we call `bhai` to estimate the german burden of HAIs. The result is another PPS object which stores burden estimates together with the data. After calling `bhai`, typing `german_pps_repr` prints an additional summary of the estimated burden:

```
german_pps_repr = bhai(german_pps_repr)
german_pps_repr
##   Country: Germany (representative sample)
##   Patients in PPS: 9,626
##   Patients with HAI: 414
##   HAIs: HAP, SSI, BSI, UTI, CDI
##
##   Cases:  478,368 (422,347 - 532,868)
##   Deaths: 16,331 (10,728 - 22,794)
##   DALY:   248,916 (175,225 - 334,351)
```

3.1 Summary tables

Summary tables for each HAI and overall estimates of the number of cases, deaths, DALYs, years lived with disability (YLD) and years of life lost (YLL) can be easily extracted from the `german_pps_repr`. `bhai.prettyTable` extracts the median, 2.5% and 97.5% quantiles for each statistic and infection and returns an easy to read summary table (see Table 1):

```
prettyTab_repr = bhai.prettyTable(german_pps_repr)
```

3.2 Summary plots

BHAI provides three functions for visualization of the result: `bhai.circleplot`, `bhai.barplot` and `bhai.strataplot`. `bhai.circleplot` and `bhai.barplot` can be used to get a comprehensive overview and comparison of the number of cases, deaths and DALYs of each HAI (Figure 2). `bhai.circleplot` plots the number of cases against the number of deaths where each HAI is plotted as a circle whose width is proportional to its DALYs. `bhai.barplot` plots the number of cases, deaths and DALYs for each disease per 100,000 population.

```
par(mfrow=c(2,2))
bhai.circleplot(german_pps_repr)
bhai.barplot(german_pps_repr, what="DALY")
bhai.barplot(german_pps_repr, what="Cases")
bhai.barplot(german_pps_repr, what="Deaths")
```

`bhai.strataplot` plots the number of cases, deaths and DALYs stratifed by age and gender. We illustrate the function with the number of cases, deaths and DALYs for all HAIs and the german representative sample. The results are shown in Figure 3.

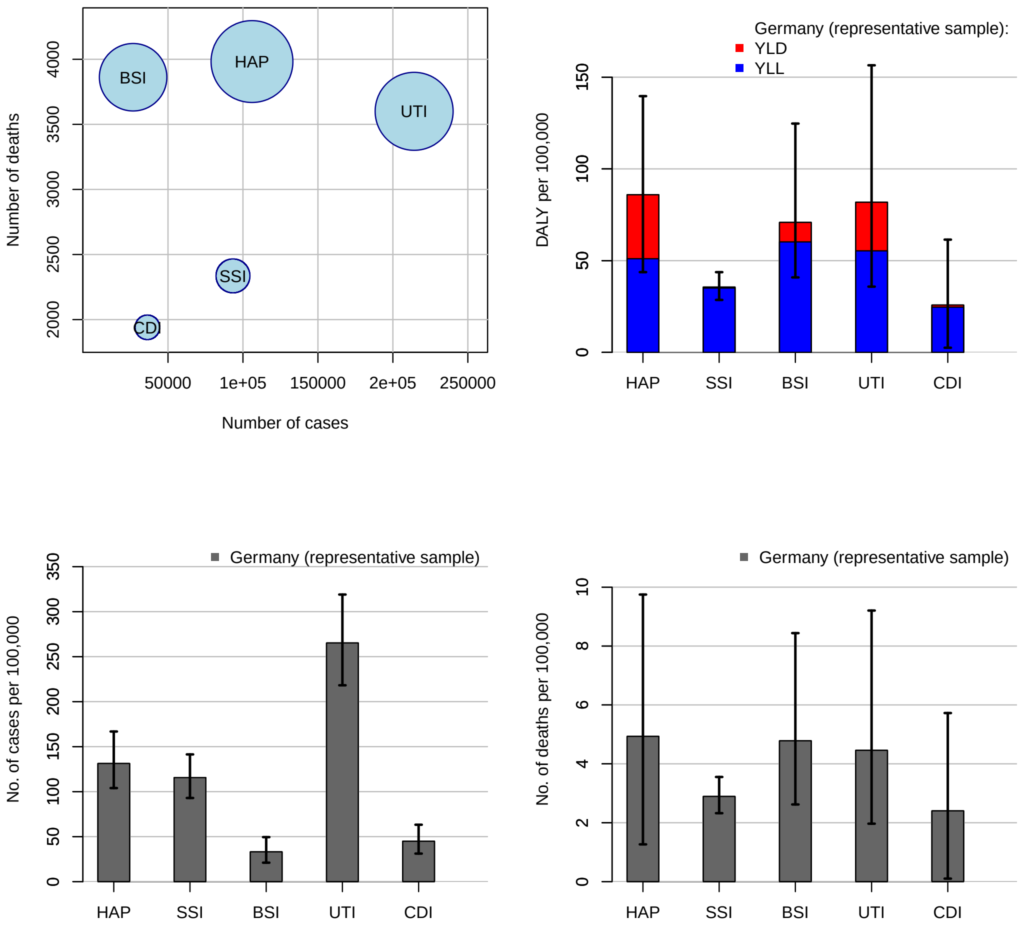

Figure 2: Results for the german representative sample 2011. Topleft: Number of cases is plotted against the number of deaths. Circles are proportional to DALYs. Topright: DALYs per 100,000 population for all HAIs. Bottomleft: Number of cases per 100,000 population for all HAIs. Bottomright: Number of deaths per 100,000 population for all HAIs.

Table 1: Summary table of the german representative sample

	Cases	Deaths	DALY	YLL	YLD
**HAP**	106,016 (83,941 - 134,720)	3,983 (1,022 - 7,874)	69,385 (35,329 - 112,801)	41,229 (10,541 - 80,805)	27,500 (15,993 - 42,607)
**SSI**	93,342 (75,043 - 114,161)	2,336 (1,877 - 2,870)	28,750 (23,091 - 35,317)	28,305 (22,740 - 34,771)	451 (340 - 582)
**BSI**	26,724 (17,002 - 39,940)	3,862 (2,116 - 6,815)	57,211 (32,997 - 100,720)	48,635 (26,709 - 87,544)	8,538 (4,598 - 15,756)
**UTI**	214,170 (176,201 - 257,636)	3,600 (1,589 - 7,435)	66,092 (28,939 - 126,382)	44,697 (19,260 - 91,830)	19,766 (8,028 - 38,826)
**CDI**	36,247 (25,161 - 51,067)	1,941 (83 - 4,623)	20,834 (2,035 - 49,632)	19,921 (876 - 48,043)	965 (185 - 2,192)
**ALL**	478,368 (422,347 - 532,868)	16,331 (10,728 - 22,794)	248,916 (175,225 - 334,351)	189,637 (126,179 - 264,577)	58,450 (39,247 - 84,031)

```
bhai.strataplot(german_pps_repr, infection="ALL", what="Cases")
bhai.strataplot(german_pps_repr, infection="ALL", what="Deaths")
bhai.strataplot(german_pps_repr, infection="ALL", what="DALY")
```

3.3 Comparison of multiple results

`bhai.barplot` enables the comparison of multiple results. In this section we illustrate this functionality using the german convenience sample and data from the EU PPS 2011. We load the *PPS* objects containing the german convenience sample and data from the european PPS and apply `bhai`:

```
# Estimate german burden with convenience sample
data(german_pps_2011_conv)
german_pps_conv = bhai(german_pps_conv)
german_pps_conv
##   Country: Germany (convenience sample)
##   Patients in PPS: 41,539
##   Patients with HAI: 1,779
##   HAIs: HAP, SSI, BSI, UTI, CDI
##
##   Cases:  513,425 (478,550 - 555,334)
##   Deaths: 19,958 (13,483 - 26,261)
##   DALY:   295,144 (214,399 - 373,503)
# Estimate EU burden
data(eu_pps_2011)
eu_pps = bhai(eu_pps)
eu_pps
```

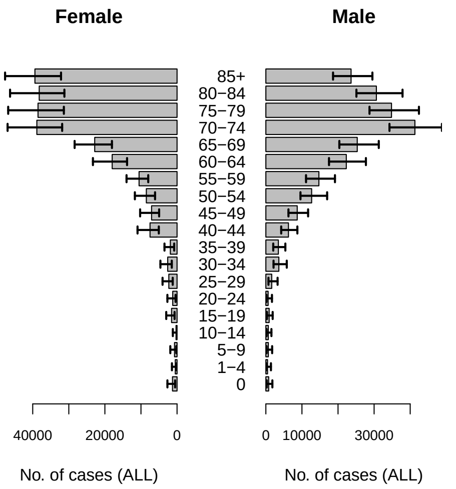

(a)

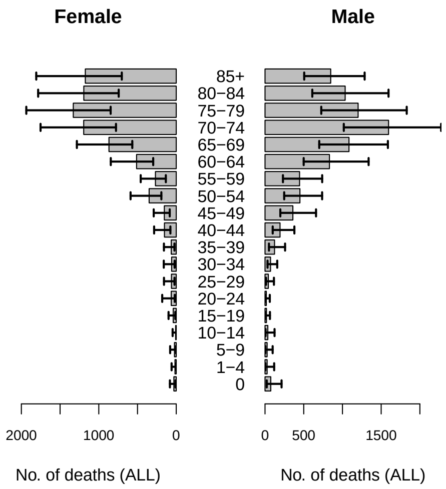

(b)

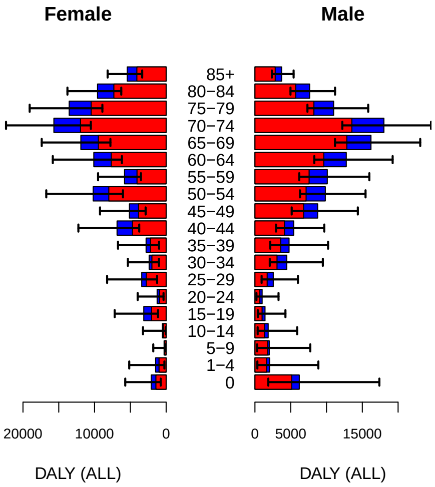

(c)

Figure 3: Visualization of the stratified number of (a) cases, (b) deaths and (c) DALYs (german representative sample 2011). YLL are shown in red, YLD in blue.

```
##   Country: European Union
##   Patients in PPS: 190,718
##   Patients with HAI: 8,878
##   HAIs: BSI, HAP, UTI, SSI, CDI
##
##   Cases:  2,362,245 (2,299,601 - 2,428,145)
##   Deaths: 77,538 (52,862 - 107,309)
##   DALY:   1,478,549 (1,119,690 - 1,904,921)
```

In order to display multiple results side-by-side we simply pass all results to `bhai.barplot` to plot DALYs, number of cases and deaths. The results can be seen in Figure 4.

```
bhai.barplot(german_pps_repr, german_pps_conv, eu_pps, what="DALY")
bhai.barplot(german_pps_repr, german_pps_conv, eu_pps, what="Cases")
bhai.barplot(german_pps_repr, german_pps_conv, eu_pps, what="Deaths")
```

4 Stratified vs. unstratified sampling

The approach of *BHAI* essentially implements a modified workflow of [1]. When applied to small sample sizes the approach by Cassini et al. overestimates the burden of HAIs because of the stratified sampling that is used. The methodological adjustments in *BHAI* were made in order to overcome this limitation and present a tool that is applicable to small and large samples. In this section we want to compare both approaches and illustrate the effect of the adjustments made in *BHAI* using simulated data with varying sample sizes. We will first apply *BHAI* to the european PPS data and then create simulated sub-samples of the data to illustrate the effect of the sample size and different choices of parameter on the results. Cassini et al. [1] used the median(LN-INT) as an estimate for the length of infection. As we discussed above, the mean might be a better estimator. Therefore we used the mean to reestimate the burden of HAIs with *BHAI* (see previous section).

The main difference of between *BHAI* and Cassini et al. is the stratification during the Monte Carlo simulations. *BHAI* samples the prevalence for each disease, estimates the number of cases and finally distributes cases into age, gender and McCabe categories according to predefined probability distributions. In contrast, the approach proposed by Cassini et al. first calculates the number of cases for each disease, age and gender and then samples the number of cases in each stratum during Monte Carlo simulations. This approach works fine with large sample sizes where the expected number of cases is greater than zero in the vast majority of strata as we can see at the example of the european PPS which is a large sample with more than 200,000 patients.

To illustrate this we apply the stratified sampling approach proposed by Cassini et al.. In order to apply the stratified sampling the denominator - i.e. the total number of patients - in each age, gender and McCabe category is needed. Moreover, the number of hospital discharges has to be stratified by age and gender as well. This data is also stored in `eu_pps`:

```
eu_pps_stratified = eu_pps
eu_pps_stratified@country = "European Union (stratified sampling)"
head(eu_pps_stratified@hospital_discharges)
##               F       M
## [0;0]   1097583 1408208
## [1;4]   1170060 1574909
## [5;9]    783914 1059224
## [10;14]  784452  921765
## [15;19] 1272099  993731
```

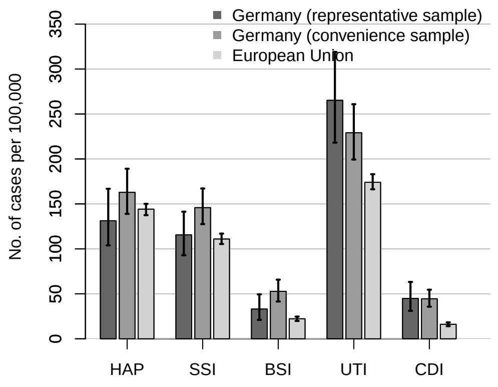

(a)

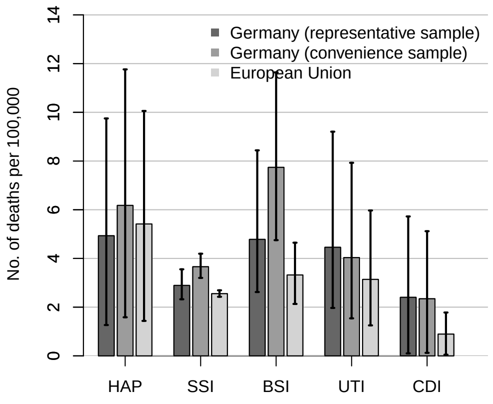

(b)

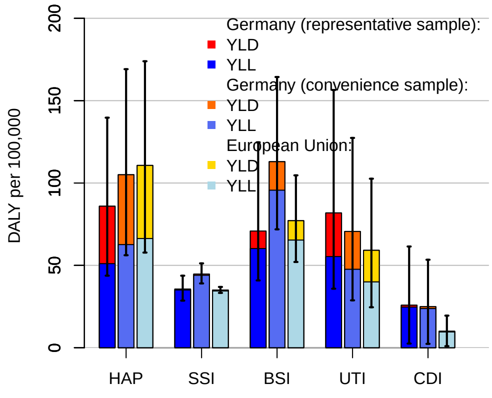

(c)

Figure 4: Comparison of the number of (a) cases, (b) deaths and (c) DALYs per 100,000 population of the german representative and convenience sample and the european sample from 2011.

```
## [20;24] 1889583  959013
names(eu_pps_stratified@num_survey_patients_by_stratum)
## [1] "NONFATAL" "RAPFATAL" "ULTFATAL"
lapply(eu_pps_stratified@num_survey_patients_by_stratum, head)
## $NONFATAL
##            F    M
## [0;0]   5037 5753
## [1;4]   1619 2027
## [5;9]    933 1250
## [10;14] 1045 1069
## [15;19] 1733 1278
## [20;24] 2680 1421
##
## $RAPFATAL
##          F  M
## [0;0]   50 54
## [1;4]   16 16
## [5;9]    8 11
## [10;14]  6  7
## [15;19] 17 19
## [20;24] 23 33
##
## $ULTFATAL
##           F   M
## [0;0]   119 125
## [1;4]    98 125
## [5;9]    72  78
## [10;14]  42  61
## [15;19]  81  96
## [20;24]  89  90
```

Setting the `stratified_sampling` option in `bhai` enables stratified sampling:

```
eu_pps_stratified = bhai(eu_pps_stratified, stratified_sampling=TRUE)
eu_pps_stratified
##   Country: European Union (stratified sampling)
##   Patients in PPS: 190,718
##   Patients with HAI: 8,878
##   HAIs: BSI, HAP, UTI, SSI, CDI
##
##   Cases:  2,275,725 (2,212,615 - 2,335,346)
##   Deaths: 76,423 (51,103 - 104,172)
##   DALY:   1,619,365 (1,208,240 - 2,080,016)
## Warning: Results were obtained using stratified sampling (not recommended)!
```

The results of the stratified (cassini et al.) and unstratified (*BHAI*) sampling approach are very similar to each other:

```
bhai.prettyTable(eu_pps_stratified, conf.int=F)[,"Cases"]
##         BSI         HAP         UTI         SSI         CDI         ALL
##   "126,211"   "691,762"   "802,425"   "568,512"    "84,229" "2,275,725"
bhai.prettyTable(eu_pps, conf.int=F)[,"Cases"]
```

```
##         BSI         HAP         UTI         SSI         CDI         ALL
##   "112,290"   "728,634"   "879,902"   "560,697"    "81,230" "2,362,245"
```

Thus we can conclude that the european data is large enough for the stratified sampling appo-rach.

5 Sample size and robustness of the estimates

The PPS samples of individual countries are much smaller than the european data ranging from 462 (Iceland) - 48,473 (United Kingdom) patients. In order to examine the effect of varying sample sizes and stratified sampling in a systematic manner we use simulated data. `sample.pps` can be used to generate simulated subsamples of a *PPS* object while keeping the prevalence fixed. We apply stratified and unstratified sampling for a wide range of sample sizes. Moreover we want illustrate the effect of the use of a prior on the age and gender distribution:

```
# Sample size for simulations
sim_survey_patients = c(seq(1000, 15000, by=1000),
                        seq(20000, 100000, by=20000),
                        seq(125000, 200000, by=25000))
# Subsample data sets from european PPS
sim_pps = sample.pps(eu_pps, num_survey_patients = sim_survey_patients)

# Run Monte Carlo simulations wit different options
sim_pps_stratified = lapply(sim_pps, bhai, summarize_strata=F,
                            stratified_sampling=T)
sim_pps_bhai = lapply(sim_pps, bhai, summarize_strata=F, use_prior=F)
sim_pps_bhai_prior = lapply(sim_pps, bhai, summarize_strata=F, use_prior=T)
```

We use `bhai.barplot` to compare the results in Figure 5. It shows a strong dependency on sample sizes when stratified sampling is used while `bhai` yields stable results even for small samples. Application of a Prior on the age and gender distribution can stabilizes DALYs when sample size is small. For small samples, the age and gender distribution observed in the PPS can be quite sparse - i.e. containing many empty strata - and there lead to an unrealistic distribution of DALYs.

```
cases_eu = eu_pps@bhai_summary$ALL$TOTAL["Cases","50%"]/eu_pps@population*100000
daly_eu = eu_pps@bhai_summary$ALL$TOTAL["DALY","50%"]/eu_pps@population*100000

par(mfrow=c(3,2))
# plot results of stratified sampling
bhai.barplot(sim_pps_stratified, infections="HAP", what="DALY",
             main="HAP (stratified sampling)",
             cols1="red", cols2="blue", names.inf=F, cex.names=0.75)
abline(h=daly_eu, col="gold", lwd=1.5, lty=2)
legend("topleft", "Estimate of full sample", col="gold", lwd=1.5, lty=2, bty="n")
bhai.barplot(sim_pps_stratified, infections="HAP", what="Cases",
             main="HAP (stratified sampling)",
             cols1="grey", names.inf=F, cex.names=0.75)
abline(h=cases_eu, col="gold", lwd=1.5, lty=2)

# plot results of bhai without Prior
bhai.barplot(sim_pps_bhai, infections="HAP", what="DALY", ylim=c(0,400),
             cols1="red", cols2="blue", names.inf=F, cex.names=0.75,
```

```
             main="HAP (bhai)")
abline(h=daly_eu, col="gold", lwd=1.5, lty=2)
bhai.barplot(sim_pps_bhai, infections="HAP", what="Cases",
             main="HAP (bhai)",
             cols1="grey", names.inf=F, cex.names=0.75)
abline(h=cases_eu, col="gold", lwd=1.5, lty=2)

# plot results of bhai with Prior
bhai.barplot(sim_pps_bhai_prior, infections="HAP", what="DALY", ylim=c(0,400),
             cols1="red", cols2="blue", names.inf=F, cex.names=0.75,
             main="HAP (bhai with Prior)")
abline(h=daly_eu, col="gold", lwd=1.5, lty=2)
bhai.barplot(sim_pps_bhai_prior, infections="HAP", what="Cases",
             cols1="grey", names.inf=F, cex.names=0.75,
             main="HAP (bhai with Prior)")
abline(h=cases_eu, col="gold", lwd=1.5, lty=2)
```

6 Concluding Remarks

This vignette was generated using the following package versions:

- R version 3.5.1 (2018-07-02), `x86_64-w64-mingw32`
- Locale: `LC_COLLATE=German_Germany.1252`, `LC_CTYPE=German_Germany.1252`, `LC_MONETARY=German_Germany.1252`, `LC_NUMERIC=C`, `LC_TIME=German_Germany.1252`
- Running under: `Windows >= 8 x64 (build 9200)`
- Matrix products: default
- Base packages: base, datasets, graphics, grDevices, methods, stats, utils
- Other packages: BHAI 0.99.0, kableExtra 1.1.0, knitr 1.24
- Loaded via a namespace (and not attached): backports 1.1.4, coda 0.19-3, colorspace 1.4-1, compiler 3.5.1, crayon 1.3.4, digest 0.6.20, evaluate 0.14, glue 1.3.1, grid 3.5.1, highr 0.8, hms 0.5.0, htmltools 0.3.6, httr 1.4.0, lattice 0.20-35, magrittr 1.5, MASS 7.3-50, Matrix 1.2-14, MatrixModels 0.4-1, mcmc 0.9-6, MCMCpack 1.4-4, munsell 0.5.0, pillar 1.4.2, pkgconfig 2.0.2, plotrix 3.7-5, quantreg 5.42.1, R6 2.4.0, Rcpp 1.0.1, readr 1.3.1, rlang 0.4.0, rmarkdown 1.14, rstudioapi 0.10, rvest 0.3.4, scales 1.0.0, SparseM 1.77, stringi 1.4.3, stringr 1.4.0, tibble 2.1.3, tools 3.5.1, vctrs 0.2.0, viridisLite 0.3.0, webshot 0.5.1, xfun 0.8, xml2 1.2.1, yaml 2.2.0, zeallot 0.1.0

References

[1] A. Cassini et al. “Burden of Six Healthcare-Associated Infections on European Population Health: Estimating Incidence-Based Disability-Adjusted Life Years through a Population Prevalence-Based Modelling Study”. In: *PLoS Med.* 13.10 (2016), e1002150.

[2] E. Colzani et al. “A Software Tool for Estimation of Burden of Infectious Diseases in Europe Using Incidence-Based Disability Adjusted Life Years”. In: *PLoS ONE* 12.1 (2017), e0170662.

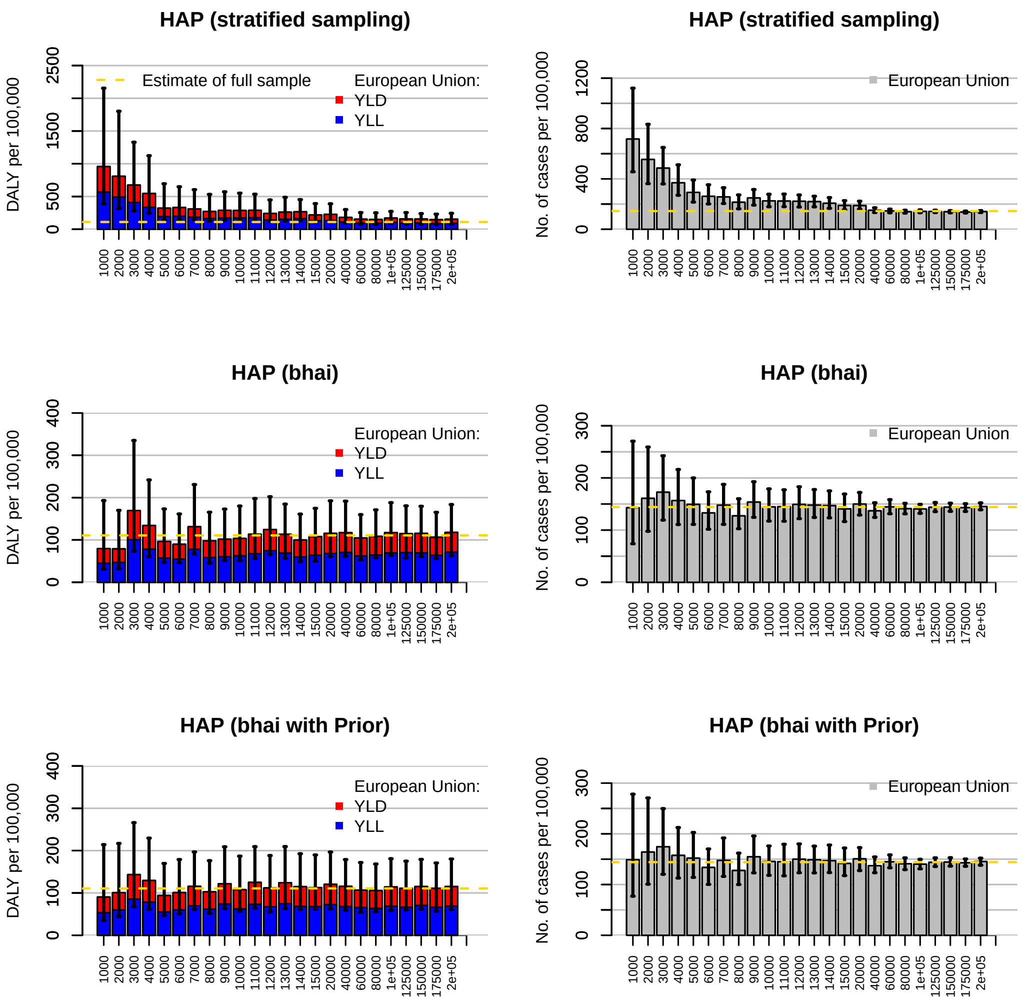

Figure 5: The effect of stratified and unstratified sampling in *BHAI* on DALYs (left) and the number of cases (right) are shown for HAP (first two rows). The results of the unstratified sampling are dependent on the sample size while `bhai` yields results which are independent of sample sizes. The bottom row shows the results of *BHAI* using an a prior. The use of a prior stabilizes DALYs when sample size is small.

[3] Niklas Willrich et al. “From prevalence to incidence - a new approach in the hospital setting”. In: *bioRxiv* (2019). DOI: 10.1101/554725. eprint: https://www.biorxiv.org/content/early/2019/02/22/554725.full.pdf. URL: https://www.biorxiv.org/content/early/2019/02/22/554725.
