# Supplementary material for: Application of a new methodology and R package reveals a high burden of healthcare-associated infections (HAI) in Germany compared to the average in the European Union/European Economic Area, 2011 to 2012
Source: Euro Surveill. 2019 Nov 14;24(46):1900135. doi: 10.2807/1560-7917.ES.2019.24.46.1900135 (PMC6864977; doi:10.2807/1560-7917.ES.2019.24.46.1900135)

PPS data

Infection	Cases in PPS
HAP	88
SSI	111
BSI	23
UTI	155
CDI	37

(1) Prevalence sampling

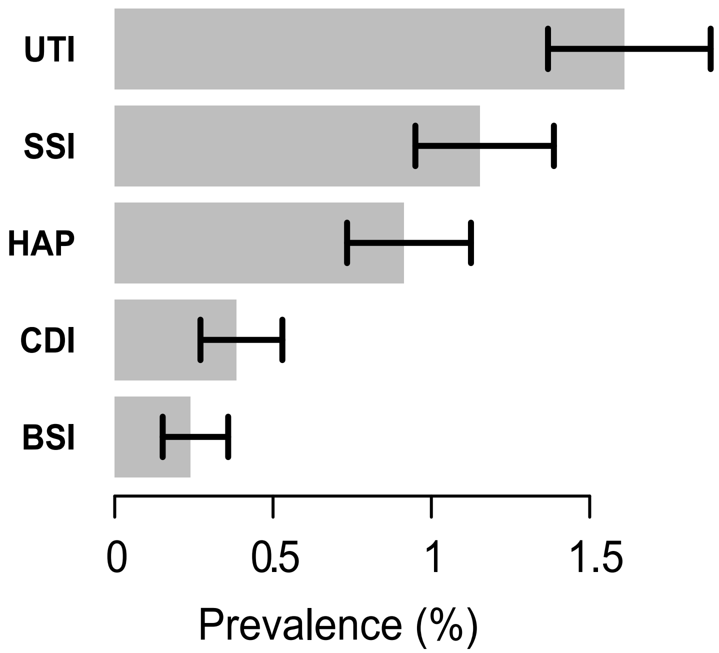

(2) Incidence estimation

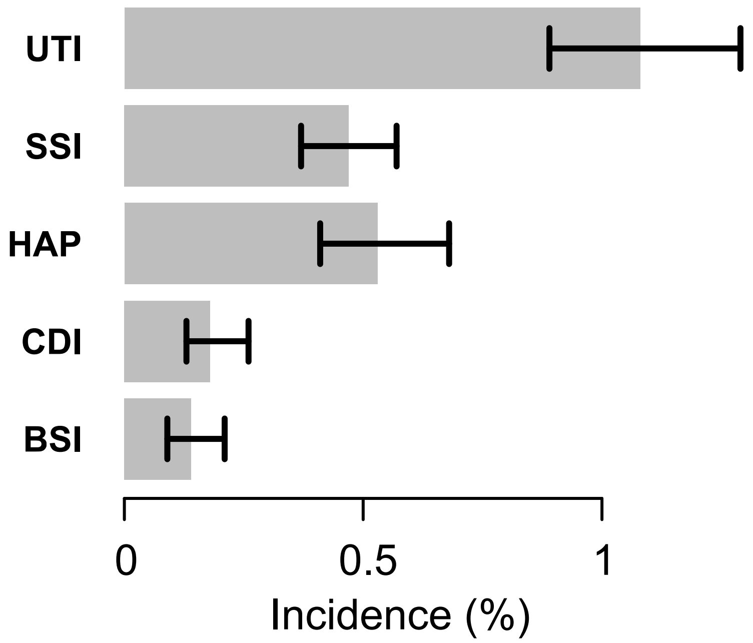

(3) Extrapolation on population

Infection	No. of cases
HAP	81,809 - 135,194
SSI	73,896 - 116,948
BSI	17,050 - 40,732
UTI	177,305 - 260,474
CDI	25,084 - 50,002

(4) Distribution of overall cases into strata

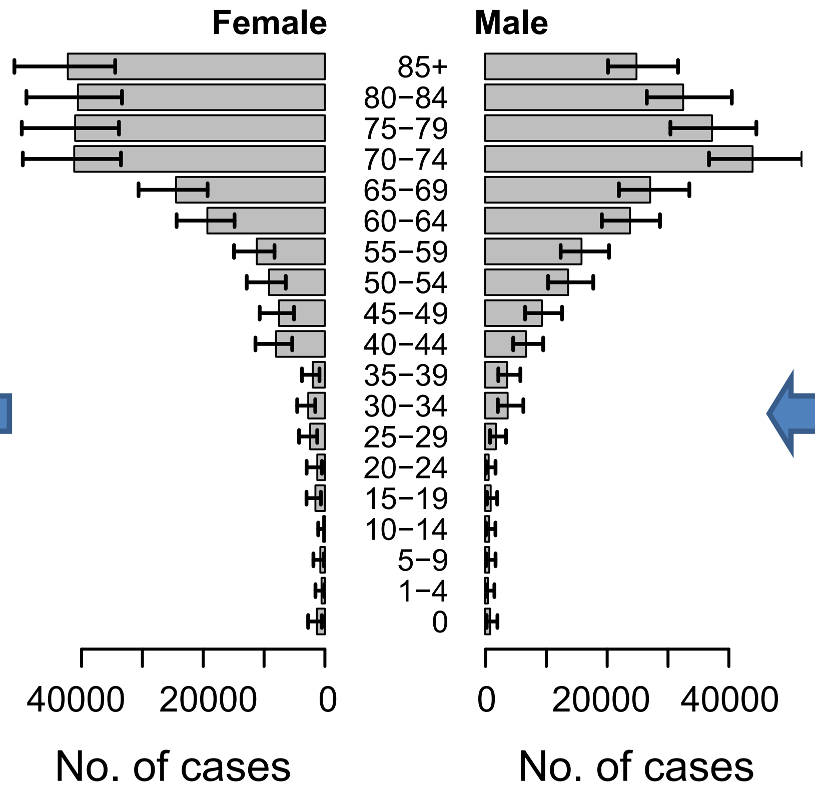

(5) Sampling of outcome trees and estimation of DALYs

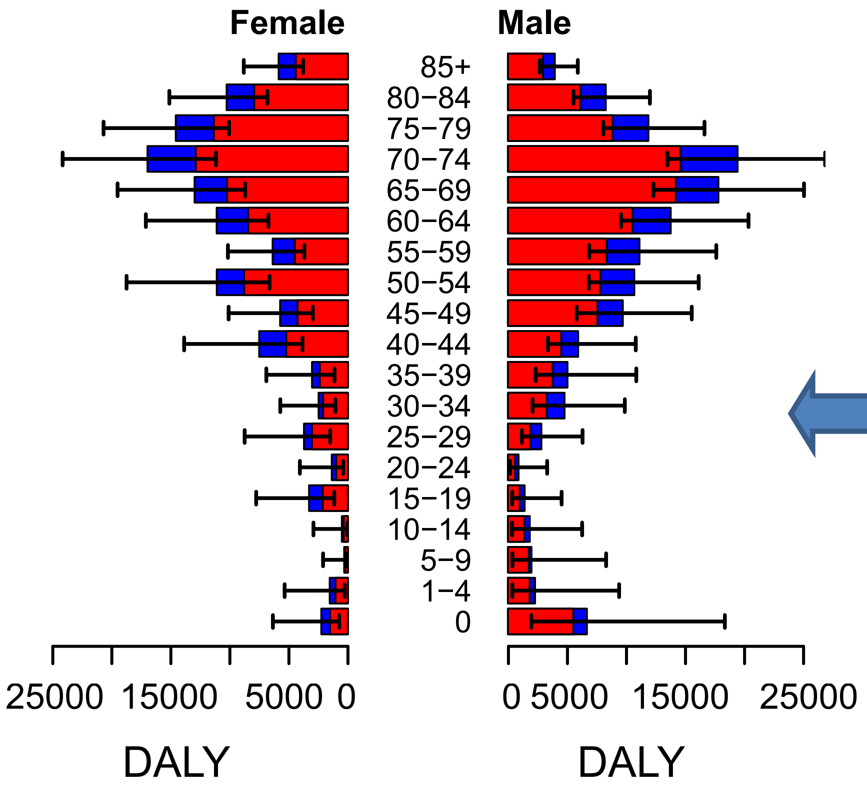

Supplement: Supplement S1 [file 1900135_ZACHER_Script.zip › BHAI.zip!/BHAI/BHAI/vignettes/workflow.pdf]
